# Mechanoelectric effects in healthy cardiac function and under Left Bundle Branch Block pathology

**DOI:** 10.1016/j.compbiomed.2023.106696

**Published:** 2023-04

**Authors:** Argyrios Petras, Matthias A.F. Gsell, Christoph M. Augustin, Jairo Rodriguez-Padilla, Alexander Jung, Marina Strocchi, Frits W. Prinzen, Steven A. Niederer, Gernot Plank, Edward J. Vigmond

**Affiliations:** a Johann Radon Institute for Computational and Applied Mathematics (RICAM), Austrian Academy of Sciences (OEAW), Altenbergerstrasse 69, Linz, 4040, Upper Austria, Austria; b Gottfried Schatz Research Center: Division of Biophysics, Medical University of Graz, Graz, 8036, Austria; c BioTechMed-Graz, Graz, 8010, Austria; d Centre Inria d’Université Côte d’Azur, Epione team, Sophia Antipolis, 06902, France; e Department of Biomedical Engineering, School of Biomedical Engineering & Imaging Sciences, King’s College London, London, SE1 7EH, United Kingdom; f Department of Physiology, Maastricht University, Maastricht, 6211, Netherlands; g Liryc, Electrophysiology and Heart Modeling Institute, Fondation Bordeaux Université, Av. du Haut Lévêque, Pessac-Bordeaux, 33600, Nouvelle–Aquitaine, France; h Université de Bordeaux, Institut de Mathématiques de Bordeaux, UMR 5251, Talence, 33400, France

**Keywords:** Computational model, Cardiac electromechanics, Mechanoelectric feedback, Stretch activated channels, Canine heart

## Abstract

Mechanoelectric feedback (MEF) in the heart operates through several mechanisms which serve to regulate cardiac function. Stretch activated channels (SACs) in the myocyte membrane open in response to cell lengthening, while tension generation depends on stretch, shortening velocity, and calcium concentration. How all of these mechanisms interact and their effect on cardiac output is still not fully understood. We sought to gauge the acute importance of the different MEF mechanisms on heart function.

An electromechanical computer model of a dog heart was constructed, using a biventricular geometry of 500K tetrahedral elements. To describe cellular behavior, we used a detailed ionic model to which a SAC model and an active tension model, dependent on stretch and shortening velocity and with calcium sensitivity, were added. Ventricular inflow and outflow were connected to the CircAdapt model of cardiovascular circulation. Pressure–volume loops and activation times were used for model validation.

Simulations showed that SACs did not affect acute mechanical response, although if their trigger level was decreased sufficiently, they could cause premature excitations. The stretch dependence of tension had a modest effect in reducing the maximum stretch, and stroke volume, while shortening velocity had a much bigger effect on both. MEF served to reduce the heterogeneity in stretch while increasing tension heterogeneity. In the context of left bundle branch block, a decreased SAC trigger level could restore cardiac output by reducing the maximal stretch when compared to cardiac resynchronization therapy. MEF is an important aspect of cardiac function and could potentially mitigate activation problems.

## Highlights

• The role of mechanoelectric feedback mechanisms in the cardiac function is explored.
• An in-silico study using a biventricular canine cardiac geometry is conducted.
• The study uses 3D-0D fluid-electromechanical framework implemented in CARP.
• Length and velocity dependence in the active tension enhances the cardiac contraction.
• Stretch activated channels introduce tension heterogeneities and regularize stretch.

## 1 Introduction

Cardiovascular diseases are the leading cause of death around the world, comprising 31% of global mortality according to the World Health Organization. The heart, which is essentially an electrically-activated mechanical blood pump, poses a challenging multiphysics problem. Different levels of mechanisms play an important role on the cardiac contraction, since cellular and subcellular processes result in changes in the tissue and organ level. Computational models can be a valuable tool in cardiology and aid in the understanding of the mechanisms of the beating heart [1]. In-silico studies have already aided the design of medical treatments, such as the development of ablation strategies for the termination of persistent atrial fibrillation [2] and the pharmacological treatment of atrial fibrillation [3] to name a few.

Several multiphysics, multiscale models have been introduced to simulate the beating heart [1]. State-of-the-art models include a coupled fluid-electromechanic framework, using the Navier–Stokes equation to model the blood flow [4], [5], [6]. Even though such detailed frameworks can provide realistic results, the identification of the flow parameters can be a challenging task in comparison to the use of 0-dimensional lumped models [7]. Recent work has coupled the 0-dimensional CircAdapt lumped model [8], [9] with the electromechanical (EM) framework implemented in the Cardiac Arrhythmia Research Package (CARP) software [10], introducing a fully coupled 3D-0D fluid-EM framework [11]. The CircAdapt model is capable of simulating cardiovascular system dynamics under a variety of physiological and pathophysiological conditions, providing more realistic boundary conditions when coupled to the EM model.

Using a multiscale framework, the effect of cellular mechanisms on organ contraction can be investigated. Of particular interest are the mechanoelectric feedback (MEF) mechanisms and their effect on heart function. The active contraction of a myocyte is stimulated through the binding of calcium to troponin C, with force generation depending on the stretch and the stretch velocity of the fibers [12]. Furthermore, the stretch of the fibers also activates ionic channels and can even result in stretch-induced depolarizations of the cell, including action potentials [13], [14]. Experimental data have shown that MEF can also have a proarrhythmic effect [15] and it can be so strong as to directly induce arrhythmias [16]; however, further research is required for the understanding of the regulatory role played by MEF. MEF mechanisms have been studied in 3D computational models on left atrial models [17], left ventricular geometries [18], [19], [20] or incorporated into a four-chamber cardiac geometry [21], however, to the best of our knowledge, never on a biventricular cardiac geometry using a fully coupled fluid-electromechanic framework.

In this paper, we employ the multiscale fluid-electrome- chanics framework in [11] to simulate the beating of canine ventricles. An active contraction model of ventricular myocytes produced tension that was dependent on length [12], while stretch activated channels introduced an additional ionic current [22]. The model was validated against physiological data. The effect of the MEF mechanisms was explored by removing the length and the stretch velocity dependence on the active contraction and the ionic channels. Additionally, the effect of the sensitivity of the stretch activated channels on the length of the fibers in the heartbeat was investigated. Finally, the model was applied to simulate left bundle branch block (LBBB), and cardiac resynchronization therapy (CRT), the typical therapy for severe cases of LBBB [23].

**Fig. 1:**
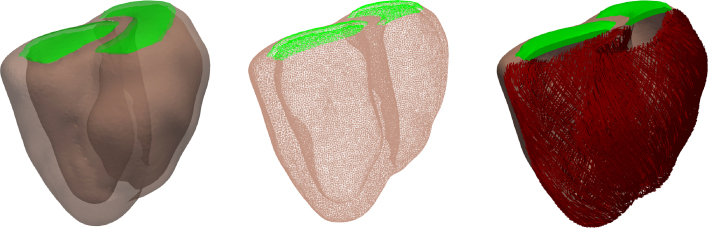
The biventricular geometry of the canine heart (left), a slice of the finite element mesh (middle) and the fibers of the model (right). The ventricular walls are shown in light brown, the layers that provide closed cavities are shown in green and the fibers in red.

## 2 Methods

### 2.1 Geometry

We used a previously developed canine biventricular geometry [11], constructed from cardiac magnetic resonance image (MRI) data obtained by a Philips Gyroscan 1.5T (NT, Philips Medical Systems, Best, Netherlands) at end-diastolic phase from a 20.6 kg mongrel dog [24]. A finite element discretization was generated using CGAL (www.cgal.org) and smoothed using meshtool [25]. The discretized geometry consisted of 111,234 points defining 557,316 tetrahedral elements with an average edge length of 1.3 mm (see Fig. 1). The fibers were constructed with a rule-based algorithm [26] with fiber angles of 60° and -60°, and sheet angles of -65° and 25° on the endocardial and epicardial surfaces respectively. Two planes covering the cavities were used to model the stiffness of the base of the heart and to provide closed cavities for the biventricular geometry, as shown in Fig. 1.

### 2.2 Computational model

#### 2.2.1 Mechanical material

The ventricular wall material was hyperelastic and nearly incompressible, and was modeled using the orthotropic material by Usyk et al. [27] and calibrated for canine data. The model reads as

(1) $$\begin{array}{c} \Psi \left(E\right)=\frac{C}{2}\left(e^{Q}-1\right)+\frac{k}{2}\ln^{2}\left(J\right),\\ Q=b_{ff}E_{ff}^{2}+b_{ss}E_{ss}^{2}+b_{nn}E_{nn}^{2}+2\left(b_{fs}E_{fs}^{2}+b_{fn}E_{fn}^{2}+b_{sn}E_{sn}^{2}\right), \end{array}$$

where Ψ is the strain energy function, J is the Jacobian of the deformation gradient tensor, E with the corresponding subscripts ff, ss and nn indicate the axial strain tensor in the fiber, sheet and normal direction respectively, and E with the corresponding subscripts fs, fn and sn the shear strain tensor in the fiber-sheet, fiber-normal and sheet-normal planes respectively. C and b with the corresponding subscripts are the passive material parameters and k is the incompressibility factor. More details can be found in [11].

The layers that provide closed cavities were modeled by a stiffer Neohookean material, to account for the rigidity of the valves, with the addition of the incompressibility penalty term

(2) $$\begin{array}{c} \Psi \left(E\right)=c_{v}\left(I_{1}-3\right)+\frac{k}{2}\ln^{2}\left(J\right), \end{array}$$

where $I_{1}$ is the first invariant of the Cauchy–Green strain tensor and $c_{v}$ is the valve material parameter.

#### 2.2.2 Electrophysiological modeling

Since the spatial discretization was too coarse for the use of the monodomain equation to simulate electrical propagation in the tissue, we used the hybrid reaction-eikonal model [28]. This method first solves the eikonal equation to determine activation times for each node x, $t_{a}\left(x\right)$ at which

(3) $$C_{m}\frac{\partial V_{m}}{\partial t}=I_{foot}\left(t-t_{a}\left(x\right)\right)+\nabla \cdot \sigma_{i}\nabla V_{m}-I_{ion}$$

where $C_{m}$ is the membrane capacitance per unit area, $V_{m}$ is the transmembrane potential, $\sigma_{i}$ is the intracellular conductivity tensor, $I_{ion}$ is the membrane ionic current, and $I_{foot}\left(t\right)$ is a stimulus current which mimics the foot of the depolarizing current occurring in tissue. At a cellular level, the Ten Tusscher ionic model [29] was used for the ventricular myocytes.

#### 2.2.3 Electromechanical coupling

The active tension model developed by the Land et al. model [12] simulated the generation of the contraction force. This model consists of 6 ordinary differential equations (ODEs) and incorporates length dependence and myocyte velocity on the generated tension and the calcium sensitivity. The generated tension, $T_{a}$, and the terms that introduce length and velocity dependence in the model are given by

$$T_{a}\left(\lambda ,\overset{˙}{\lambda }\right)=h\left(\lambda \right)\frac{T_{ref}}{r_{s}}\left(\left(\zeta_{s}+1\right)S+\zeta_{w}W\right),$$

$$\frac{d\zeta_{s}}{dt}=A_{eff}\overset{˙}{\lambda }-c_{s}\zeta_{s}$$

$$\frac{d\zeta_{w}}{dt}=A_{eff}\overset{˙}{\lambda }-c_{w}\zeta_{w}$$

(4) $$h\left(\lambda \right)=\max\left(0,h^{\prime }\left(\min\left(\lambda ,1.2\right)\right)\right)$$

$$h^{\prime }\left(\lambda \right)=1+\beta_{0}\left(\lambda +\min\left(\lambda ,0.87\right)-1.87\right)$$

$$\frac{dCaTRPN}{dt}=k_{TRPN}\left({\left(\frac{{\left[{\text{Ca}}^{2+}\right]}_{i}}{{\left[{\text{Ca}}^{2+}\right]}_{T50}}\right)}^{n_{TRPN}}\times \right)\left(\left(1-CaTRPN\right)-CaTRPN\right)$$

$${\left[{\text{Ca}}^{2+}\right]}_{T50}={\left[{\text{Ca}}^{2+}\right]}_{T50}^{ref}+\beta_{1}\left(\min\left(\lambda ,1.2\right)-1\right)$$

where λ is the stretch ratio, $\overset{˙}{\lambda }$ is the velocity, $T_{ref}$ is the maximal active tension at resting length, $r_{s}$ is the steady state duty ratio of the presented three-state crossbridge model, $\zeta_{s}$ and $\zeta_{w}$ are the distortions at the pre-powerstroke state W and force generating post-powerstroke state S, $c_{s}$ and $c_{w}$ are the decay rates of the distortions $\zeta_{s}$ and $\zeta_{w}$ respectively, CaTRPN is the fraction of troponin C bound with calcium, $k_{TRPN}$ is the binding rate, $n_{TRPN}$ is the cooperativity of the binding rate, ${\left[{\text{Ca}}^{2+}\right]}_{i}$ is the intracellular calcium, ${\left[{\text{Ca}}^{2+}\right]}_{T50}^{ref}$ is the half activation point for the calcium and troponin C binding, $\beta_{0}$ and $\beta_{1}$ introduce the length dependence in the induced tension and the activation calcium, and $A_{eff}$ is velocity dependence coefficient.

The calcium sensitivity of the induced tension model introduces a one-way coupling with the Ten Tusscher model, which provides the intracellular calcium. Since myocytes branch and are not pure cylinders, tension is distributed between the fiber and the transverse directions, with the latter reaching up to 50% of that in the fiber direction [30]. We thus assumed that 40% of tension acted in the transverse direction.

Additionally, we considered the presence of stretch activated channels (SACs), for which we use the simple model by Kohl and Sachs [22] for the generating current

(5) $$I_{SAC}=G\frac{V_{m}-E_{SAC}}{1+Ke^{-\alpha \left(\lambda -1\right)}},$$

where G is the stretch activated conductance, $E_{SAC}$ is the reversal potential, α and K are scaling factors, λ is the myocyte fiber stretch. The generated current was added to the ionic currents in the Ten Tusscher model, providing a contribution of mechanics to the ionic model. SAC channels were adjusted to obtain an action potential at a stretch of 10% in single cell, and rescaled to ensure that no early depolarizations occur at a stretch of 8%–10% (see model parametrization section in supplementary material).

#### 2.2.4 Circulation model

The 0D CircAdapt lumped model [9] was considered for the simulation of the hemodynamics and cardiovascular system. CircAdapt consists of a variety of modules (such as tubes, valves, chambers) that are interconnected with flows, introducing a total of 26 ODEs. The 0D ODE model is strongly coupled with the 3D PDE system [11].

#### 2.2.5 Boundary conditions

Neumann pressure boundary conditions were applied in the enclosed cavity surfaces of the ventricles (endocardium and interior surface of the valves), which were obtained from the CircAdapt model [11]. Robin type boundary conditions are applied on the pericardium (including the outer surface of the valves), corresponding to springs with variable stiffness that restricted motion in the normal direction [31]. Near the apex, spring stiffness was increased, by applying a penalty scale, following the results in Strocchi et al. [31]. The stiffness varied from the apex to the base with a maximum of 0.001 kPa/μm.

Electrophysiologically, His-Purkinje activation was approximated by the simultaneous activation of the entire endocardial surfaces of both ventricles.

#### 2.2.6 Numerical solution

The proposed electromechanical model was numerically solved using the coupled 3D-0D finite element framework implemented in the Cardiac Arrhythmia Research Package (CARPentry) [11], [32], [33]. The cardiac cycle length was 600 ms, corresponding to 100 beats per minute [34]. The activation of the right atrium was at the onset of the beat, with an inter-atrial delay of 40 ms [34]. The atrioventricular delay was set to 130 ms [34], [35], and no interventricular delay was considered. CircAdapt model parameters were tuned to closely approximate the end-diastolic volume of the canine geometry. All parameters of the model can be found in the supplementary material.

To determine initial electrical conditions, isolated cells were first paced until steady state was reached, ignoring stretch. Next, using the backward displacement method [36], the unloaded stress-free geometry was obtained. Finally, the coupled 3D-0D simulations were performed until a maximum of ten simulated heartbeats, wherein steady state was obtained, using the Newton iterative method with a maximum number of 50 iterations and a relative error reduction of the residual of 10^−6^ [11].

## 3 Results

### 3.1 Model validation

**Fig. 2:**
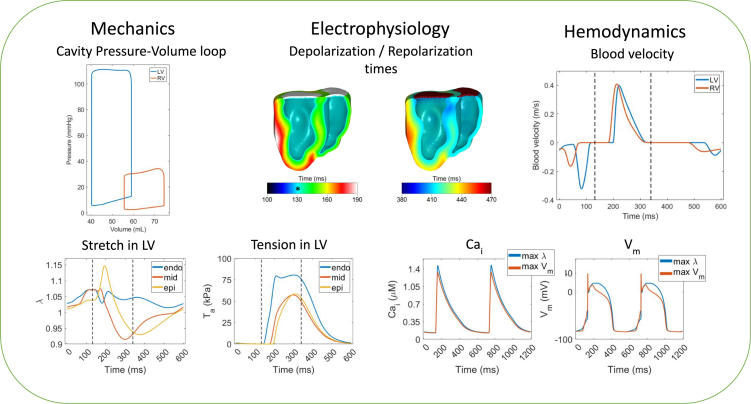
The mechanical, electrophysiological and hemodynamic results of the base model. Abbreviations: LV: Left Ventricle, RV: Right Ventricle, $Ca_{i}$: Intracellular calcium concentration, and $V_{m}$: Transmembrane voltage. Top left: the pressure–volume loop of the LV and the RV. Top middle: the depolarization and repolarization times, where * indicates the onset of ventricular depolarization. Top right: the inflow (negative values) and outflow (positive values) velocities of the LV and RV. In bottom left, the stretch and tension of selected radially collinear nodes on the endocardium, midwall and epicardium of the anterior wall of the LV over a heartbeat. The dashed black lines indicate the cardiac systole. In the bottom right, the intracellular calcium and transmembrane potential of two heartbeats are shown for the nodes with the largest stretch and largest potential before the application of the stimulus (Nodes II and III in the supplementary material).

We first needed to establish a base model for our simulations. Fig. 2 shows a summary of the fluid-electromechanics simulation results. The final tuning and calibration of the model occurred on the organ level with the parameters as summarized in the supplementary material.

The pressure volume loop was used to calibrate the model against physiological data. In particular, the end-diastolic volume (EDV) is comparable with the one obtained from the canine cardiac MRI scan. The geometry had a cavity volume of 57 mL for the left ventricle and 75.3 mL for the right ventricle, which closely matched, with an error of 2 mL from our model, as the simulated EDVs were 59.1 mL for the LV and 74.5 mL for the RV. Experimental data [37] indicate that the shape of the Pressure–Volume loop (PV-loop) of the LV is visually similar for the case of 100 beats per minute simulated by our model.

Our model approximated the peak and end-diastolic pressure as 111.2 mmHg/12.7 mmHg for the LV, and 34.2 mmHg/6.5 mmHg for the RV, which lie within the physiological ranges for canine hearts [38], [39]. The LV end-diastolic pressure was slightly larger than reported [39]; however, the pacing technique used in our work is different since both endocardia activate at the same time. The stroke volume of both the LV and RV of our model were 18.8 mL, which are within measured ranges [39], [40], [41].

The electromechanical activation times derived from electrocardiograms (ECGs) for normal canines have also been considered for the validation of our model. Specifically, the inter-atrial and PR interval were chosen to match experimental data in the literature [34], [35]. The simulated QRS complex duration of our model is 75 ms, which is within the reported values for normal canine hearts in [35]. The duration of the isovolumetric contraction and the ejection phases were 68 ms and 139 ms respectively, which also lie within the physiological range [42].

### 3.2 Mechanoelectric effects

**Fig. 3:**
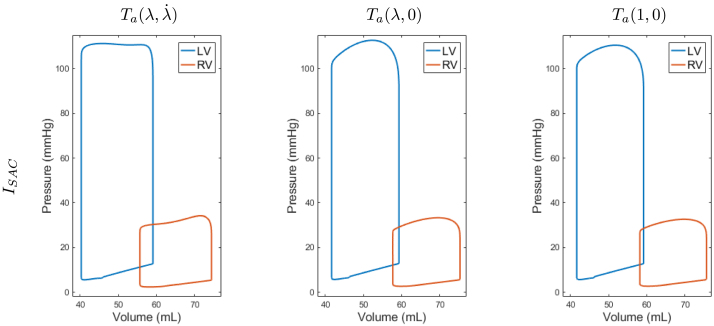
Pressure–Volume loops of the simulated heartbeats with SACs $I_{SAC}$, and for different combinations of the MEF mechanisms. Active tension depended on both stretch and velocity ($T_{a}\left(\lambda ,\overset{˙}{\lambda }\right)$), only on the stretch ($T_{a}\left(\lambda ,0\right)$), or without any stretch dependence ($T_{a}\left(1,0\right)$). Comparable results are obtained without SACs.

We explored the effect of MEF described in Section 2.2.3 on the simulated heartbeat. The mechanisms considered were the length and velocity dependence of the tension development in the active contraction model [12], and the influence of SAC currents on electrophysiology [22]. The simulations were performed using the parameters as described in Section 3.1 and selectively removing subsets of the MEF mechanisms. To eliminate the contribution of the SACs, we simply exclude the $I_{SAC}$ from the ionic currents. To remove the velocity dependence in the active tension model, we set the parameter $A_{eff}$ to zero and we write it as $T_{a}\left(\lambda ,0\right)$, while to additionally remove the length dependence we set the parameters $\beta_{0}=\beta_{1}=0$, and we denote it as $T_{a}\left(1,0\right)$. When we removed the dependence of the active contraction model on the velocity, the induced tension became larger, as reflected by Eqs. (4). The further removal of the stretch dependence resulted in a reduced, yet rather high tension as reflected by $T_{ref}$. Thus, in these cases, we had to retune the parameter $T_{ref}$ to 70 in order to reduce peak pressure to a value comparable to the one of our base model. The resulting PV-loops for all six combinations appear in Fig. 3, and a summary of the peak pressure, the stroke volume, the maximal rate of change of the ventricular pressure and the ejection duration for each ventricle is presented in Table 1.

The results show that there is a strong impact of the velocity dependence of the active tension on the response of the model. In particular, a big decrease of 27 ms for the LV and 19 ms for the RV in the duration of the ejection phase and a decrease of 0.82 mmHg/ms and 0.15 mmHg/ms for the LV and RV, respectively, in the maximal rate of change of pressure are the dominant changes in comparison to the model with both stretch and velocity dependence. Furthermore, the end-systolic pressure is smaller in the LV (96.3 mmHg vs. 94.4 mmHg) and larger for the RV (23.0 mmHg vs. 23.9 mmHg). A decrease of 1 mL in the stroke volume also appears for both ventricles. The peak pressure is comparable up to 1 mmHg, as obtained by tuning $T_{ref}=70$ in the active tension model for the model without the velocity dependence.

**Table 1:** The effect of the MEF mechanisms with SACs, $I_{SAC}$, on peak pressure (PP), stroke volume (SV), maximal rate of change of the ventricular pressure (dP/dt), and ejection phase duration for the left and right ventricles. Comparable results are obtained without SACs.

		PP (mmHg)		SV (mL)		max dP/dt (mmHg/ms)		Ejection duration (ms)	
		LV	RV	LV	RV	LV	RV	LV	RV
$I_{SAC}$	$T_{a}\left(\lambda ,\overset{˙}{\lambda }\right)$	111.2	34.2	18.8	18.8	2.85	0.90	139	165
	$T_{a}\left(\lambda ,0\right)$	112.7	33.3	17.7	17.6	2.03	0.75	112	146
	$T_{a}\left(1,0\right)$	110.5	32.6	17.5	17.5	1.90	0.71	119	152

Further removing the stretch dependence from the tension has a smaller impact in comparison to the velocity dependence. There is a small increase in the ejection duration phase (7 ms for the LV and 6 ms for the RV), while the maximal rate of change of the pressure is further decreased by 0.13 mmHg/ms and 0.04 mmHg/ms for the LV and RV, respectively. A small change appears for the end-systolic pressure (94.4 mmHg vs. 93.1 mmHg for the LV and 23.9 mmHg vs. 23.4 mmHg for the RV), and no appreciable changes in the stroke volume. The peak pressure changes by 2 mmHg and 0.7 mmHg for LV and RV in comparison to the model with $T_{a}\left(\lambda ,0\right)$ as tuned by using $T_{ref}=70$, but still remains comparable to the model with both the velocity and stretch dependence $T_{a}\left(\lambda ,\overset{˙}{\lambda }\right)$, with differences up to 1.6 mmHg.

While the stretch and velocity dependence had an impact on the PV-loops, no appreciable differences appear from the presence of the stretch activated channels $I_{SAC}$.

**Fig. 4:**
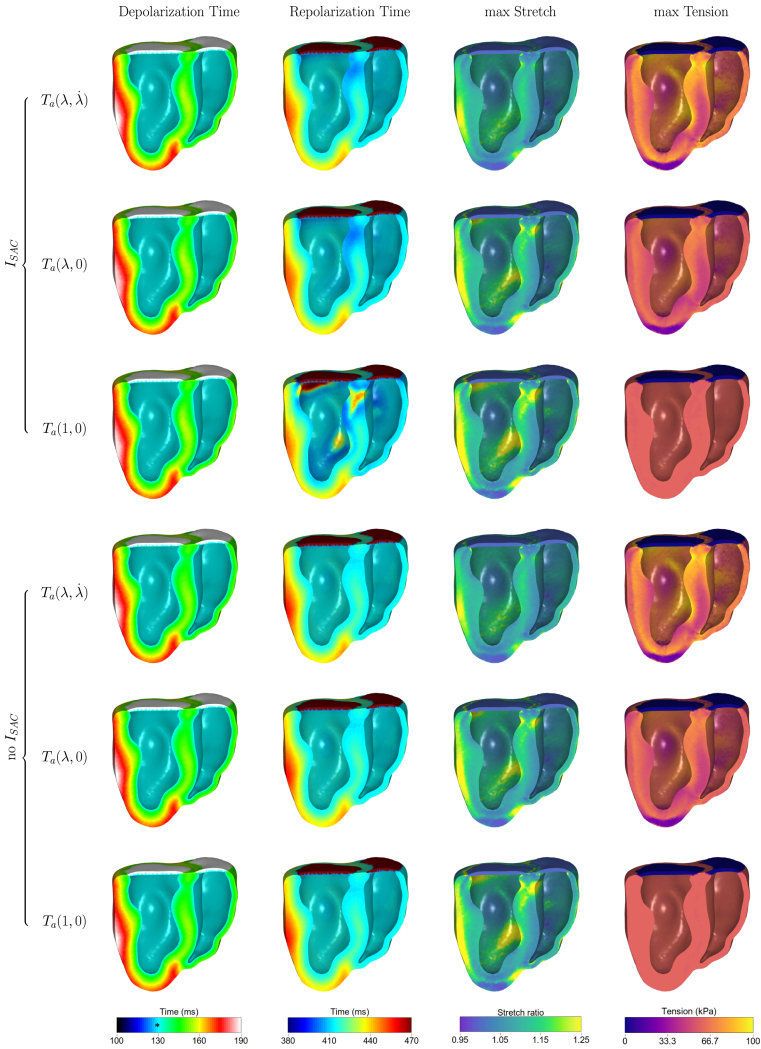
The effect of the MEF mechanisms on the depolarization and repolarization times, the maximum stretch ratio and the maximum active tension over the final heartbeat. The ∗ indicates the onset of the ventricular depolarization at 130 ms.

Next, we looked at the MEF effect on the depolarization and repolarization times of the ventricular walls, as well as the maximum stretch and the maximum tension over a heartbeat (see Fig. 4). The presence of SACs led to a rise of the potential from the resting state before the application of the stimulus (as shown in the supplementary material for the cellular EP response), however there were no premature excitation areas. In the absence of SACs, no depolarization of the resting potential was observed before the application of the stimulus.

A large difference appears in repolarization times, where early repolarization areas appear in the endocardium of both ventricles and the septum in the cases of the presence of both MEF, or without the velocity dependence in the induced tension model (see also supplementary material for the effect on the cellular EP). This effect is more evident and accompanied with prolonged repolarization areas in the absence of the length and velocity dependence in the active tension. No differences are observed in the absence of SACs, as expected.

On the other hand, the length and velocity dependence of the induced tension had a bigger effect on the maximum stretch ratio λ and tension over a heartbeat, similar to what has been observed in other works on a rat model [43], [44]. In particular, in the case of $T_{a}\left(\lambda ,\overset{˙}{\lambda }\right)$, smaller areas of large maximum stretch appear, accompanied with a heterogeneous tension, as shown from the median and quartile values in Table 2 and in the histograms in the supplementary material. The absence of the velocity dependence leads to larger stretch in the LV and septum and a smaller heterogeneity in the tension distribution within the ventricular walls (with an interquartile range of 0.07 vs. 0.08 for the stretch ratio and 16.4 kPa vs. 10.3 kPa for the tension). This effect is magnified with the absence of this MEF, with a rather homogeneous tension and a larger area with high stretch ratio (interquartile range of 0.11 for the stretch ratio and 0.06 kPa for the tension). In the presence of SACs, this area matches the area of the late repolarization times, as expected from the electromechanical coupling of our model.

**Fig. 5:**
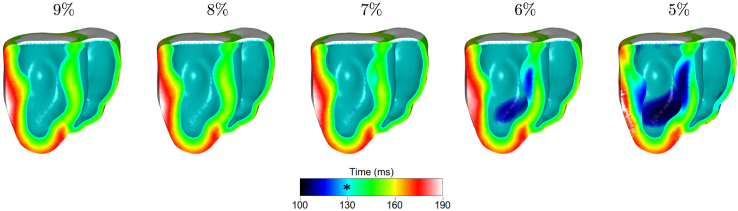
The effect of SAC trigger level on depolarization time. The ∗ indicates the onset of the ventricular depolarization at 130 ms. Premature depolarization areas became more pronounced in the septum and LV as the trigger level was decreased.

Due to the uncertainty in the sensitivity of the SACs to stretch, we explored the sensitivity of the model to the choice of SACs parameters. The effect of reducing the trigger level can be seen in Fig. 5.

**Table 2:** The median and first and third quartiles (Q1 and Q3) of the simulated maximum stretch ratio and tension over a heartbeat for the different combinations of MEF mechanisms.

	$I_{SAC}$			No $I_{SAC}$		
	$T_{a}\left(\lambda ,\overset{˙}{\lambda }\right)$	$T_{a}\left(\lambda ,0\right)$	$T_{a}\left(1,0\right)$	$T_{a}\left(\lambda ,\overset{˙}{\lambda }\right)$	$T_{a}\left(\lambda ,0\right)$	$T_{a}\left(1,0\right)$
Max stretch						
Median	1.10	1.11	1.11	1.10	1.11	1.11
[Q1:Q3]	[1.06 : 1.13]	[1.08 : 1.16]	[1.07 : 1.18]	[1.06 : 1.13]	[1.08 : 1.16]	[1.08 : 1.18]
Max tension						
Median	63.4	58.0	59.7	63.2	57.9	59.8
[Q1:Q3]	[54.9 : 71.3]	[52.3 : 62.6]	[59.4 : 60.0]	[54.8 : 71.2]	[52.1 : 62.5]	[59.5 : 60.1]

The reduction of the trigger level resulted in more depolarized areas before the application of the stimulus in the endocardium at 130 ms. Before 130 ms, the volume of the heart is increasing due to venous blood return. As the sensitivity of the SACs in the stretch increased, the most stretched regions depolarized, notably the septum.

For 9% and 8% trigger levels, no premature excitations occurred, as expected from the rescaling of the SACs parameters; however, further reducing the trigger level resulted in areas of early depolarizations. At 7% depolarizations occur 10 ms before the application of the stimulus. This effect become stronger by increasing the sensitivity of the SACs, with evident depolarization areas at 6% and 5%, in which the earliest depolarization times are 23 ms and 37 ms, respectively, before sinus activation. An increase in the end-diastolic pressure of the LV was observed as well, which reached 13.8 mmHg at 5% level. Reducing further the trigger level resulted in early contractions and asynchronous beats, and no steady state could be reached even after 10 simulated beats.

### 3.3 Application to Left Bundle Branch Block

Left Bundle Branch Block (LBBB) is a condition in which the electrical signal does not propagate from the atrioventricular node to activate the left ventricle through the His-Purkinje fiber system; thus, the ventricular electrical activation is introduced only in the RV, resulting in a longer activation time compromising cardiac output [23]. To model LBBB, the sinus stimulus was only applied to the lower third of the RV septal endocardium, following reported isochrones [45]. All other parameters were assumed to remain the same as in the base model simulation setup in Section 3.1, and the 10% trigger level was used for the SACs. The numerical simulation considered an acute response of the model, performing only two beats with the application of the LBBB stimulus and using the base model as an initial condition (Section 3.1).

**Fig. 6:**
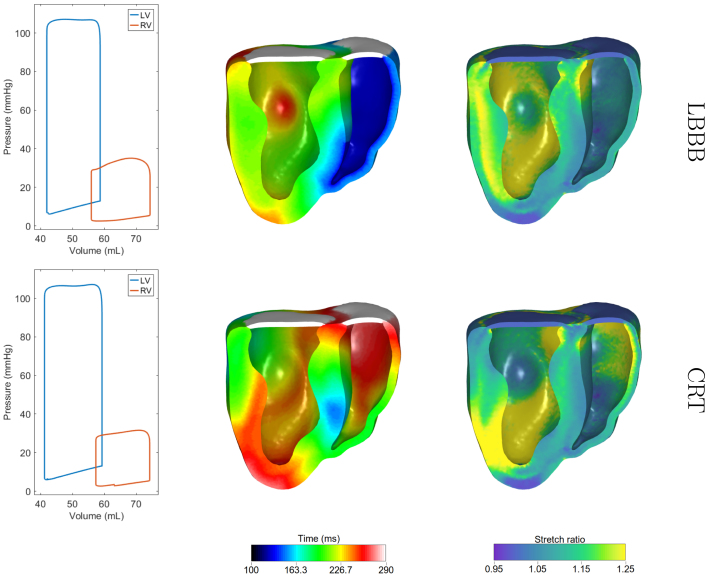
PV-loops (left column), depolarization times (middle column), and maximum stretch (right column) under LBBB conditions (top row), and after the application of CRT (bottom row). The onset of the ventricular depolarization is at 130 ms for the LBBB and at 120 ms for the CRT.

The simulated PV-loop along with the depolarization times and the maximum stretch over a heartbeat appear on the top row of Fig. 6. The results are summarized in 3. There was a decrease in the peak pressure of the LV by 4.1 mmHg and a difference of 2 mL in the stroke volume, while minor differences appear in the end-diastolic volume and pressure in comparison to the base model in 3.1. The activation of the LV is significantly longer due to the electrical wave having to propagate through the myocardium to excite the LV. The duration of the simulated QRS was 138 ms, which is comparable to the available data in the literature [35]. Our model shows that the LBBB pathology appears to have a much larger impact on the maximum stretch over a heartbeat, which seems to be much larger in nearly the entire LV compared to the baseline case.

The MEF feedback effect on the simulated LBBB pathology was explored and results are presented in detail in the supplementary material. The simulation of the LBBB followed the same approach as for the model with all MEF, and the initial condition was chosen as the corresponding model with the MEF under consideration (as obtained in Section 3.2). The PV-loops show some differences among the six models, with the largest decrease in the peak pressure of the LV being 6.4 mmHg in the case of no $I_{SAC}$ and $T_{a}\left(\lambda ,\overset{˙}{\lambda }\right)$, and the smallest 0.8 mmHg for the model with $I_{SAC}$ and $T_{a}\left(1,0\right)$. Similarly, the largest and smallest decreases in the stroke volume appeared on the same models as 3 mL and 0.5 mL respectively. The presence of the $I_{SAC}$ had some effect on both the peak pressure and the stroke volume of the LV, with differences between 2.1 mmHg–2.4 mmHg and 0.7 mL–1 mL. The impact of the SACs is further evident when exploring the depolarization times (see supplementary material), with areas activating faster than in the model without this MEF. This effect was stronger in the absence of the velocity dependence, or in the case of no stretch and velocity dependence in the active tension, where the areas of maximal stretch were gradually larger in the LV and the top part of the septum.

**Table 3:** The peak pressure (PP), end-diastolic volume (EDV), stroke volume (SV), end-diastolic pressure (EDP) and maximal rate of change of pressure (max dP/dt) of the LV for the LBBB pathology and the application of CRT in comparison to the baseline model.

	PP (mmHg )	EDV (mL )	SV (mL )	EDP (mmHg )	max dP/dt (mmHg/ms )
LBBB	107.1	58.7	16.7	12.7	2.39
CRT	107.2	59.3	18.0	12.9	2.47
Baseline	111.2	59.1	18.8	12.7	2.85

A typical treatment of the LBBB condition is cardiac resynchronization therapy, in which a lead is placed in the coronary sinus and delivers electrical pacing pulses to the LV epicardium in order to trigger more synchronous ventricular contraction [23]. To simulate CRT, we introduced two point stimulae, one on the epicardium of the LV and one near the right ventricular apex, which were activated with an atrioventricular delay of 120 ms and no interventricular delay following typical empirical device programming [46]. Considering all the remaining parameters unchanged, two beats were simulated using the CRT stimulus, with the LBBB model results as an initial condition. The simulated PV-loop and depolarization times appear at the bottom row of Fig. 6 and the results are summarized in 3.

The application of CRT increased the stroke volume of the LV by 1.3 mL. The stroke volume as well as the end-diastolic volume and pressure obtained are comparable to the baseline model. These results are within the observed experimental values [39], where the authors paced from the base of the LV. The maximal rate of LV pressure increased from the LBBB pathology, however it did not reach the baseline model. The activation of the ventricles for CRT started at time 120 ms and completed by 328 ms. Our model predicts that CRT reduces the maximum stretch near the LV base and the RV apex; however, large stretches remained in the lower part of the LV and near the base of the RV.

The effect of the MEF mechanisms in CRT simulations is shown in detail in the supplementary material (Figures 9 and 10). In all cases, an improvement from the corresponding LBBB pathology simulations was observed, regardless of any MEF mechanisms present. The changes in the PV-loops between the different models were comparable to the ones presented in Section 3.2. Minor differences are observed regarding the depolarization of the myocytes among all four models.

As an alternative treatment method for LBBB, the sensitivity of the SACs was explored in the presence of the pathology. Detailed data are supplied in the supplementary material. As the SAC trigger level was decreased, the ventricles became more synchronous and the stroke volume and peak pressure of the LV were gradually increasing. At the 5% level, the ventricles contracted nearly synchronously: The stroke volume increased to 18.8 mL from 16.7 mL and the peak pressure increased by 5.1 mmHg to 112.2 mmHg, which are both improved in comparison to the case of a 10% trigger level, and comparable with CRT. Contrary to the application of the CRT, the maximal stretch ratio appeared to decrease in the entire ventricular walls as the trigger level of the SACs was decreasing.

## 4 Discussion

In this paper, we use a multiscale fluid-electromechanical canine biventricular model using the 3D-0D coupled finite element framework described in [11] to study the effect of MEF on cardiac function. The orthotropic material by the Usyk model [27] described the passive properties of the ventricles, while the eikonal formulation modeled electrical propagation in the tissue. At the myocyte level, the Ten Tusscher model [29] was considered for the electrophysiological behavior. Different MEF mechanisms were introduced to take an active part in the model, similar as to what has been previously done in the atria [17]. Length dependence on the induced tension, which is the cellular level mechanism responsible for the Frank–Starling law, correlates the hemodynamic load with the generated force. This mechanism is described by the availability of troponin-C binding sites for calcium. The velocity of the cell shortening also regulated tension developed, as did the intracellular calcium concentration. The model also considered ionic channels which open in response to stretch, and have been shown to be able to trigger action potentials [13], [14]. The addition of these channels in the model introduces a strong coupling of the mechanical on to the electrical physics.

In the presence of MEF mechanisms, the model was validated against experimental canine data [35], [38], [39], [40], [41], [42], and MRI data. The validation process used data obtained from the simulated pressure and volume over a heartbeat, as well as visual comparison of the PV-loop shape of the LV with experimental data in the literature [37]. A previous study [47] showed computational data from a normal canine heart which was different from the one presented here, in terms of volumes, with an RV smaller than the LV. The study simulated a beagle heart [48] and physiological properties of canine hearts vary based on the body weight [49]. Similar to the study [47], we found tension greatest in the endocardium with the midwall and epicardium comparable, and found endocardial stretch to be less than epicardial, with midwall stretch the greatest. Quantitatively there are differences, part of which can be attributed to discretization differences of the mechanical meshes as we used 557,316 tetrahedral elements and they used 172 Hermite-based hexahedra. The magnitude of the anterior wall fiber stretch of our model is comparable with the experimental study on canines [50] and a similar trend is observed, with a larger stretch closer to the endocardium and a delayed response from the epicardium before the opening of the aorta. The maximal rate of ventricular pressure for the LV was found to be comparable with experimental normal canine heart data [51].

### 4.1 MEF effects on the model

The effects of the MEF mechanisms on the simulated heartbeat were evaluated by performing simulations with no MEF mechanisms or a subset of them. The presence of the length-dependence on the induced tension has a very strong effect on the stroke volume and the duration of the ejection phase. This is the expected behavior, following the Frank–Starling law, since the greater hemodynamic input and corresponding stretch of the myocytes, the stronger the contraction of the heart. The effect is evident in the magnitude of the stress in the LV during the ejection phase, as shown in the supplementary material. The magnitude of the produced fiber stress values are closer to the experimental values in [52] (see supplementary material).

On the other hand, the stretch activated channels appear to have a minor impact on the contraction of the heart. Small changes occur at the closure of the mitral valve and before the opening of the aortic valve, at the end-diastolic pressure of the ventricles. They also had no impact on the distribution of the strains and the stresses in the model. This is consistent with atrial simulations showing no acute response with SACs [17], and also with single cell simulations which showed that SAC effects took minutes to appear [53]. The presence of the SACs had a stronger effect on the activation of the myocytes, as irregular action potentials and early activations appeared, which are supported by experimental results [14]. Depending on the sensitivity of the channels to the stretch, early depolarizations could result in premature ventricular contractions. These ectopic firings would occur close to natural sinus activation, resulting in a fusion of the QRS on the ECG. In particular, at trigger level of 5%, the ejection phase begins nearly 10 ms earlier in the LV, in comparison to the 10% level. This behavior can also alter the mechanical response of the heart, by increasing the end-diastolic pressure and decreasing the stroke volume. Increasing further the sensitivity of the channels on the stretch resulted in asynchronous beats and in an unsteady heartbeat. The arrhythmogenesis induced by stretch has been observed in experimental studies [14].

### 4.2 Homogenization

The effect of MEF was to homogenize the maximum stretch while at the same time leading to greater stress heterogeneity. This might not be unexpected as the mechanisms, SACs and the Frank–Starling law, are both functions of stretch and not stress. The tension dependence on prestretch had a modest effect on stretch and a small effect on strain. The tension dependence on shortening velocity was more important, having a much larger effect on both the maximum stress and maximum stretch. MEF reduced overall stretch as well as its heterogeneity while increasing septal stress, especially near the base. Thus, it appears that MEF serves to more equitably distribute stretch which is important since excessive stress will destroy a myocyte. We assumed uniform properties, but there may be natural gradients and local remodeling which will also effect the stress/strain distribution.

### 4.3 MEF effect on LBBB

When simulating the LBBB pathology, the MEF mechanisms have a comparable impact as on the healthy heart. In particular, the length dependence of the active contraction enhances the cardiac output and the duration of the ejection phase of the model. However, LBBB still results in a reduction of the cardiac output when compared against the corresponding healthy case, as the stroke volume and the peak pressure are decreased.

The presence of the SACs results in a significant change the contraction of the heart and the cardiac output due to the abnormally large stretch that stems from the LBBB. Its significance also lies in the propagation of the electrical signal of the heart, where areas in the LV are activated due to stretch. Despite seeing these activated areas, the effect of the LBBB dominates for a trigger level of 10%. Again, while the acute response may be small, a response may build over a longer time period [53].

SACs have been characterized as a potential arrhythmogenetic mechanism [14], and may result in a number of irregularities in the action potential of the myocytes, such as altered repolarization, early afterdepolarizations, or premature excitation. In the presented model, arrhythmogenic behavior observed is altered repolarization, and premature excitation patterns, which become more prominent for smaller trigger levels of the SACs.

### 4.4 Significance of SACs

The inclusion of the SACs introduces minor changes for a healthy heart simulation. These small changes might be useful for personalization of the cardiac models, however their impact does not appear to be significant. On the other hand, for pathologies such as LBBB, where Purkinje activation is blocked, SACs can play a key role in the synchronization of the ventricles.

While stretch activated channels might induce arrhythmias, further understanding of the mechanism may also be useful as treatment for electrical signal malfunctions of the heart, as noted in [17]. In particular, increasing the sensitivity of the ionic channels that are activated by stretch might lead to the resynchronization of the heart without the use of a pacemaker. Our research shows that increasing SAC sensitivity improved the ejection fraction and the cardiac output of the heart, while reducing the maximal stretch on the ventricles. On the other hand, using CRT improves the cardiac function, but at a cost of increased maximal stretches near the LV apex. Thus this method could potentially be used in order to replace existing effective therapies, such as CRT.

On the other hand, the SACs might induce arrhythmogenesis, by altering the action potential of the myocytes and prematurely exciting the cells [14]. Other MEF mechanisms, such as the stretch-induced myofilament calcium release, in combination with the SACs might result in an increased risk for arrhythmogenesis under specific strain patterns [54], while other MEF mechanisms, such as the stress assisted diffusion, might decrease this risk by competing with the effects of SACs [55]. The treatment of such arrhythmias involve the use of drugs for blocking of the stretch activated channels, which introduce the electrical dysrhythmia of the heart [14], [56].

### 4.5 Limitations

Computer models and simulations can give insights on the role of mechanoelectric feedback in the cardiac function. The results of this work can complement but not replace experimental testing.

In this study, the ventricular myocytes are considered homogeneous, while the cardiac wall is quite heterogeneous, consisting of healthy myocytes, fibroblasts and adipose tissue among other cell types. We have also assumed that material and functional properties are constant over the ventricles, which is probably not the case, but there is no data to inform the model at the level of detail required.

Due to the size of the computational geometry, the use of the reaction-Eikonal model instead of the more physiologically accurate monodomain or bidomain models was considered, thus imposing the given propagation velocity in the model. A finer geometry that allows the use of the monodomain model would consist of millions of elements, and would greatly increase the computational cost of the model. We have previously demonstrated that this approximation produces errors within acceptable limits [28].

A one-way coupling of the ionic Ten Tusscher model with the active contraction is considered, without the alteration of the calcium due to the binding with the troponin C in the ionic model. The introduction of a two-way strong coupling is part of our ongoing work. Preliminary results appear in the supplementary material, where we propose a way of strongly coupling the two models and we estimate the impact of the stretch as well as the impact of the SACs on stretch patterns obtained by our 3D model.

Finally, the LBBB pathology affects the cardiac output; however, we do not consider any alterations in the blood circulation as described by the CircAdapt model. This is acceptable as we are concerned with differences in cardiac output with respect to the MEF mechanisms.

## CRediT authorship contribution statement

**Argyrios Petras:** Study design, Analysis, Writing, Editing and approving the final version of the manuscript. **Matthias A.F. Gsell:** Study design, Analysis, Writing, Editing and approving the final version of the manuscript. **Christoph M. Augustin:** Study design, Analysis, Writing, Editing and approving the final version of the manuscript. **Jairo Rodriguez-Padilla:** Study design, Analysis, Writing, Editing and approving the final version of the manuscript. **Alexander Jung:** Study design, Analysis, Writing, Editing and approving the final version of the manuscript. **Marina Strocchi:** Study design, Analysis, Writing, Editing and approving the final version of the manuscript. **Frits W. Prinzen:** Study design, Analysis, Writing, Editing and approving the final version of the manuscript. **Steven A. Niederer:** Study design, Analysis, Writing, Editing and approving the final version of the manuscript. **Gernot Plank:** Study design, Analysis, Writing, Editing and approving the final version of the manuscript. **Edward J. Vigmond:** Study design, Analysis, Writing, Editing and approving the final version of the manuscript.

## Declaration of Competing Interest

The authors declare that they have no known competing financial interests or personal relationships that could have appeared to influence the work reported in this paper.

## Appendix A Supplementary data

MMC S1 The parameters of the models and further analysis appear in the supplementary material.
