# Supplementary material for: Mechanoelectric effects in healthy cardiac function and under Left Bundle Branch Block pathology
Source: Comput Biol Med. 2023 Apr;156:106696. doi: 10.1016/j.compbiomed.2023.106696 (PMC10040614; doi:10.1016/j.compbiomed.2023.106696)
Supplement: MMC S1 — The parameters of the models and further analysis appear in the supplementary material. [file mmc1.pdf]

Supplementary material

The supplementary material consists of six parts: the parameter choices for the model, the effect of the mechanoelectric feedback (MEF) mechanisms on the electrophysiology (EP) and on the mechanical response of the model, the sensitivity analysis of the stretch activated channels, the effect of the MEF on the Left Bundle Branch Block (LBBB) pathology and on the Cardiac Resynchronization Treatment (CRT) treatment.

Parameter	Value	Unit	Description
General			
ρ_b	1050	kg/m^3	Blood density
t_c	0.6	s	Cardiac cycle length
Tubes			
A_t^{wall}	274 (AO), 141 (AP), 58 (VC), 85 (VP)	mm^2	Wall thickness
l_t	350 (AO), 280 (AP), 400 (VC), 200 (VP)	mm	Vessel length
k_t	8 (AO), 8 (AP), 10 (VC), 10 (VP)	-	Stiffness exponent
Chambers			
V_c	49.5 (LV), 26.3 (LA), 70.0 (RV), 23.6 (RA)	mL	Cavity volume
V_c^{wall}	71.6 (LV), 14.0 (LA), 32.8 (RV), 14.0 (RA) 30.0 (SV)	mL	Wall volume
Δt_c^{act}	0.13 (LV), 0.04 (LA), 0.13 (RV), 0.0 (RA)	s	Activation time delay at each beat
Valves			
A_v^{open}	400 (AO), 600 (MV), 400 (PV), 800 (TV) 400 (PO), 400 (SO)	mm^2	Cross-sectional area
Periphery			
Δp_{py}^{ref}	2.2 (Pu), 10 (Sy),	kPa	Blood pressure drop
q_{py}^{ref}	85 (Pu), 85 (Sy),	mL/s	Reference flow
r_{py}	3 (Pu), 7.2 (Sy),	-	Resistance scaling factor

Table 1: The parameters of the CircAdapt model. The abbreviations mean: Aorta (AO), Arteria Pulmonalis (AP), Venae Cavae (VC), Venae Pulmonales (VP), Left Ventricle (LV), Left Atrium (LA), Right Ventricle (RV), Right Atrium (RA), Septum (SV), Aortic Valve (AO), Mitral Valve (MV), Pulmonary Velve (PV), Tricuspid Valve (TV), Pulmonary Outlet (PO), Systemic Outlet(SO), Pulmonary circulation (Pu) and Systemic circulation (Sy).

1 Model parametrization

The computational model uses the coupled 3D-0D electromechanical framework by Augustin et al. [1]. This model uses CircAdapt, a 0D lumped model in order to model the circulation of the

system. The parameters chosen for this model are summarized in table 1. Any parameter that does not appear in table 1 is considered as in [1]. The ventricular wall volumes, the septum volume and the ventricular chamber volumes were directly measured on the undeformed geometry. The cross-sectional areas for all the valves add up to the valve surface area in the geometry and are within the physiological ranges for a canine heart as indicated in the Handbook of Cardiac Anatomy, Physiology and Devices [4]. The rest of the CircAdapt parameters were tuned to match the end-diastolic volume of the geometry and the physiological ranges detailed in the Model validation section (Section 3.1).

The remaining parameters are summarized in table 2. Eikonal parameters and conduction velocities were tuned to match the duration of the QRS complex from the available literature [7] and echocardiography data [3], and are similar to values found in the literature [8]. The lids covering the ventricles were considered non-conducting, with conductivities set to zero.

The Stretch Activated Channels (SACs) were tuned to 10% trigger level at the reference parameters obtained by [12] (using $\alpha = 75$). Due to the larger stretches observed from our model, we rescaled the stretch sensitivity α in the SACs to 33, to ensure that no early depolarizations occur for stretch between 8%-10%.

Parameter	Value	Unit	Description
Material			
C	1.7	kPa	Stiffness scaling of myocardium
k	650	kPa	Bulk modulus
c_v	1.0	MPa	Stiffness scaling of lids
The remaining passive material parameters are taken by [1]			
Electrophysiology			
(v_f, v_s, v_n)	$(0.42, 0.28, 0.14)$	m/s	Eikonal velocity of the myocardium
(g_f, g_s, g_n)	$(0.21, 0.08, 0.08)$	m/s	Electrical conductivities of the myocardium
Ionic model			
The parameters are taken by [13]			
Active tension			
T_{ref}	90	-	Reference tension
$[Ca^{2+}]_{T50}^{ref}$	0.8	-	Reference half activation point for Calcium
β_0	0.7	-	Length dependence parameter
A_{eff}	15	-	Velocity dependence parameter
The remaining parameters are taken by [5]			
Stretch Activated Channels			
α	33	-	Scaling factor
K	150	-	Scaling factor
E_{SAC}	-20	mV	SAC reversal potential
G	0.09	mS/cm^2	Stretch activated conductance at 10% trigger level
Activation times			
RA	0	ms	Activation of Right Atrium
AA	40	ms	Interatrial activation delay
AV	130	ms	Atrioventricular activation delay

Table 2: The parameters of the model.

2 Effect of MEF on cellular EP

To explore the effect on the cellular EP, we chose four different nodes that satisfy the following criteria: the node with the largest difference in intracellular calcium Ca_i, between the model with

both MEF, and the model without any MEF (Node I), the node with the largest transmembrane potential before the stimulus (Node II), the node with the largest stretch before the application of the stimulus (Node III), and the node with the smallest stretch before the stimulus (Node IV). All four nodes were chosen according to the results obtained by the model that includes both MEF (shown in blue) and is compared against the results from the models with different combinations of MEF, as shown in figure 1.

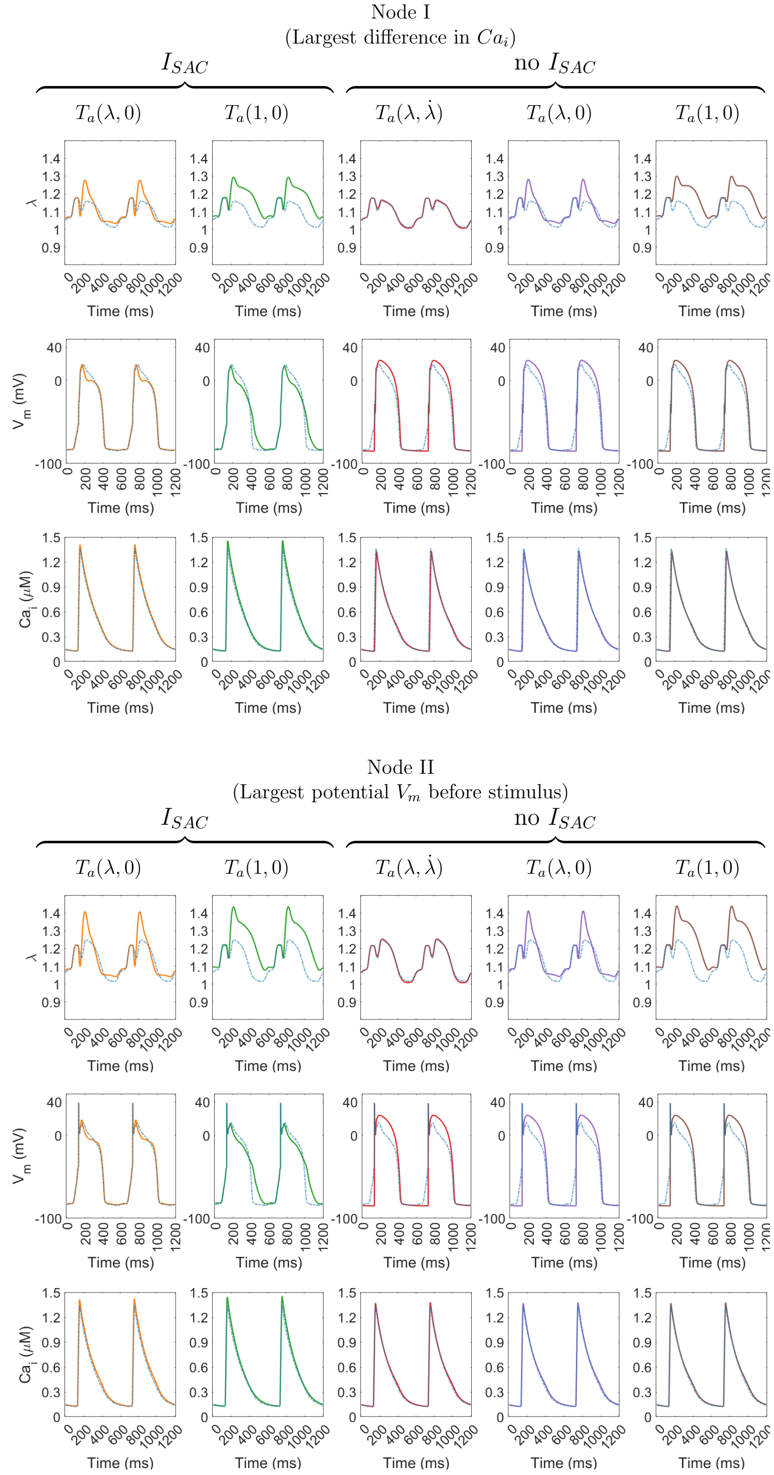

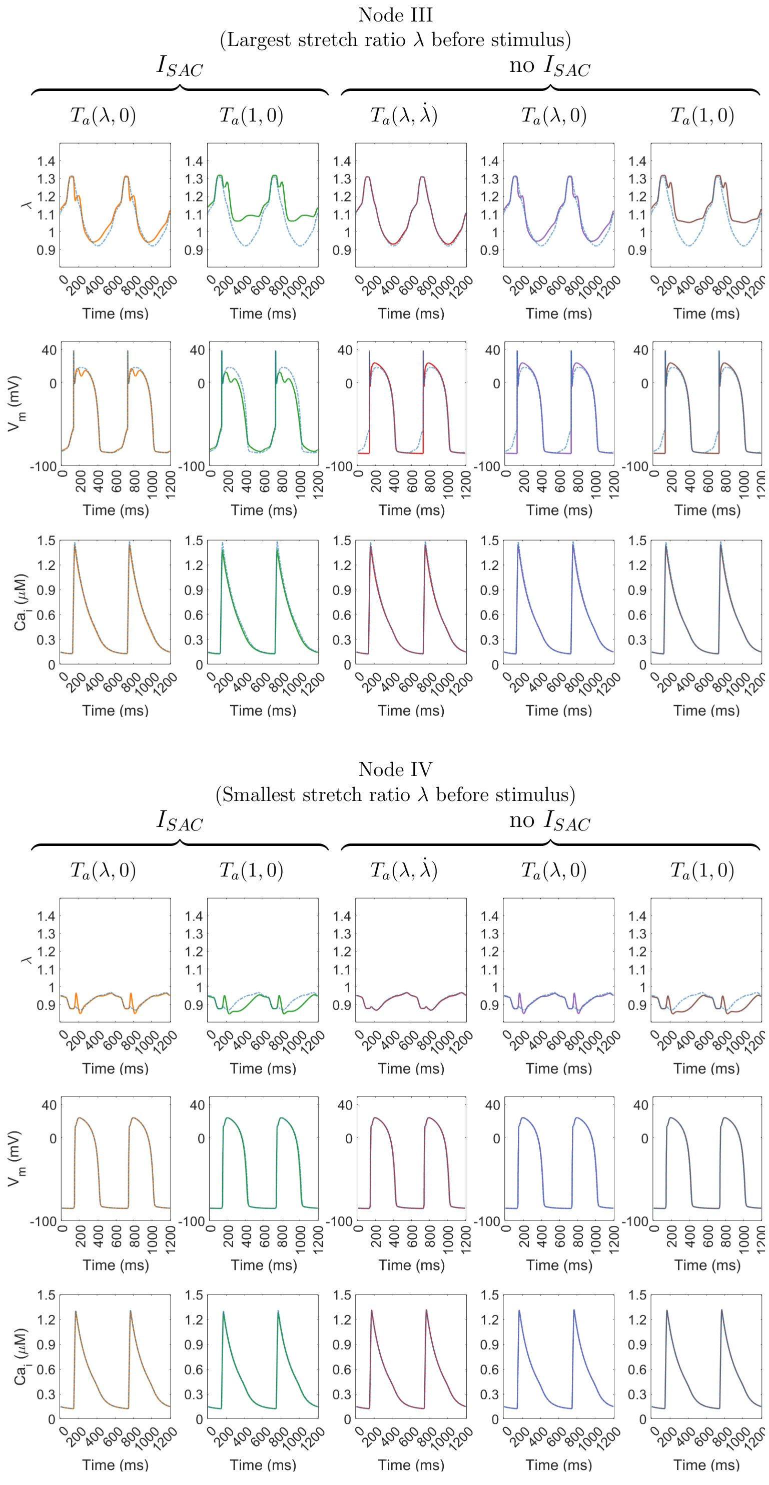

Figure 1: The stretch ratio λ, transmembrane potential V_m and intracellular calcium Ca_i of four different nodes over two heartbeats for different combinations of MEF in comparison to the model with both MEF (in blue). The nodes have been chosen based on the model with both MEF mechanisms, and Node I according to the difference to the model without any MEF.

We observe that the SACs may result in early increases of the transmembrane potential from its resting state as well as in early repolarizations. The absence of the velocity dependence in the active tension leads to larger stretch, which in turn leads to even earlier repolarization times. This effect is even more prominent in the case of independence of the induced tension in the length and velocity. On the other hand, minimal changes appear in the case that the stretch remains low, as observed on Node IV.

The potential and the calcium remain unchanged to the stretch in the absence of SACs, as expected from the lack of the mechanical feedback on Ca_i (weak coupling only).

3 Effect of MEF on mechanical response

In this section the effect of the MEF is also explored on the mechanical response of the model. At first, we show the distribution of the maximal, end systolic and end diastolic stretch ratio over a heartbeat in figure 2 and the maximal and end-systolic tension over a heartbeat in figure 3. As detailed in section 3.2 of the paper, the dependence of the tension on the length and the velocity results in a larger distribution and heterogeneity in the tension, which reduces the heterogeneity in the stretch. The SACs appear to have a minimal effect on the distribution of the stretch and tension.

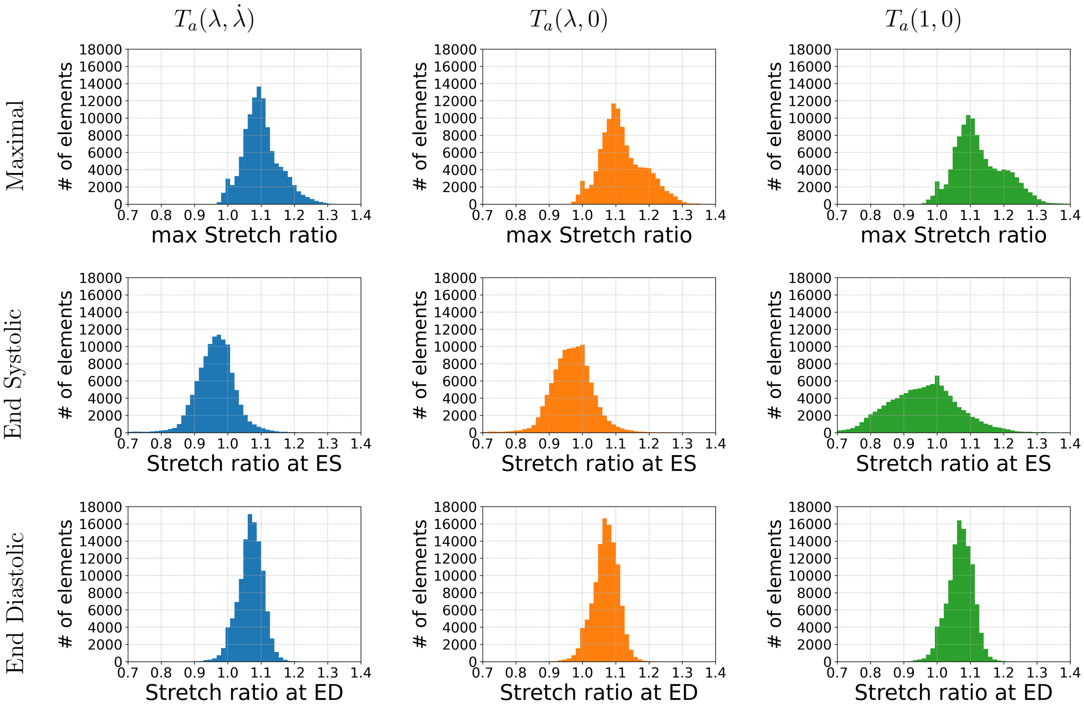

Figure 2: The distribution of the maximal, end systolic and end diastolic stretch ratio along a heartbeat for different combinations of MEF in the presence of I_{SAC}. Comparable results are obtained in the absence of SACs.

Similar to [10], the stress and strain of the myocardium in a cross section of the papillary muscle level of the LV is investigated. In particular, a slice of the LV is extracted, and the stress and strain median, first and third quartiles are displayed in figures 4 and 5. The stress remains unaffected by the presence of the SACs. On the other hand, the presence of the length and velocity dependence in the active tension introduces some changes in the interquartile range of the two tensors.

In particular, in the presence of both length and velocity dependence, the peak axial stresses occur at the end of the ejection phase, while the peak is shifted to within the ejection phase for the case of length dependence or no dependence on the stretch. Additionally, small interquartile

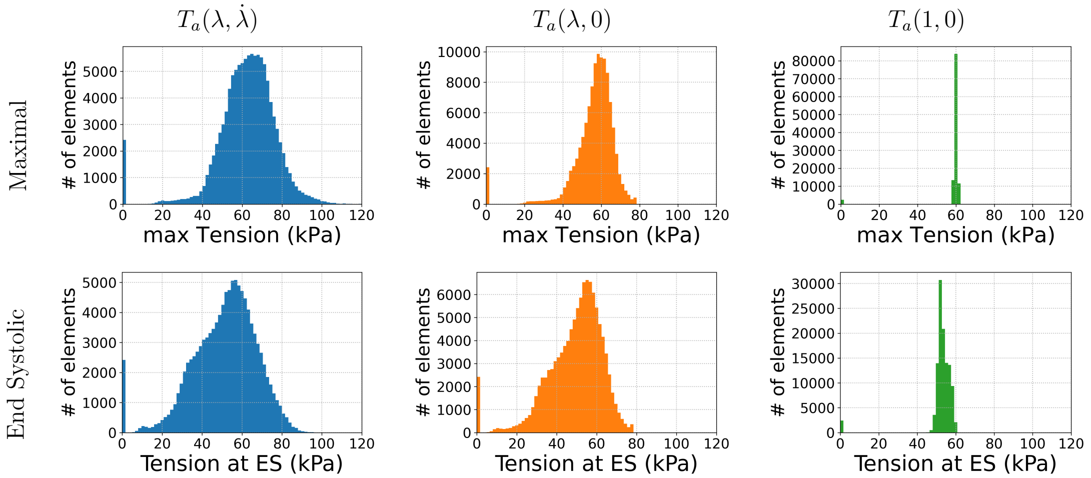

Figure 3: The distribution of the maximal and end systolic tension along a heartbeat for different combinations of MEF in the presence of I_{SAC}. Comparable results are obtained in the absence of SACs.

range differences appear for the rotational stresses. The magnitude of the stress along the fiber direction is comparable to the one calculated by [10].

Regarding the strain tensor, the results appear to be similar for all six models, regardless of the presence of the MEF mechanisms. A reduction of the strain in the fiber direction is observed in the presence of length dependence at the beginning of the ejection phase, which becomes larger when including the velocity dependence in the induced tension. The interquartile range of the rotational strains slightly decreases when including the length and velocity dependence. Finally, when comparing against the experimental results from [10], the simulated fiber strains lie within the experimental range.

4 Sensitivity of SACs

Following the sensitivity analysis of the trigger level of the SACs described in Section 3.2 of the paper, the effect of the SACs on the pressure-volume loops of the different levels was explored, and no appreciable differences appear among the different models.

5 Left Bundle Branch Block

Additional simulations were performed for the Left Bundle Branch Block pathology by removing each of the MEF or both of them. The effect of the MEF on the PV-loops and the depolarization times appear in figures 6 and 7.

Note that by removing the SACs, an observed difference of 0.7 mL in stroke volume and 2.2 mmHg in peak pressure appears among all models. The largest effect with the LBBB pathology appears in the case of length and velocity dependence in the active tension and in the absence of SACs, where the stroke volume reduction is 3 mL and the peak pressure reduction is 6.4 mmHg in comparison to the healthy model in section 3.2 of the main paper. There is a great reduction in the effect of the LBBB in the absence of velocity dependence, where only 0.5 mL difference appears in the stroke volume in the presence of SACs. This happens due to the larger stretch that occurs for these models, which is reflected in the depolarization patterns, as seen in figure 7.

5.1 Effect of sensitivity of SACs on LBBB

The sensitivity of the SACs as a potential treatment of the LBBB pathology is also investigated. Figure 8 shows the pv-loops, the depolarization times as well as the maximal stretch ratio for

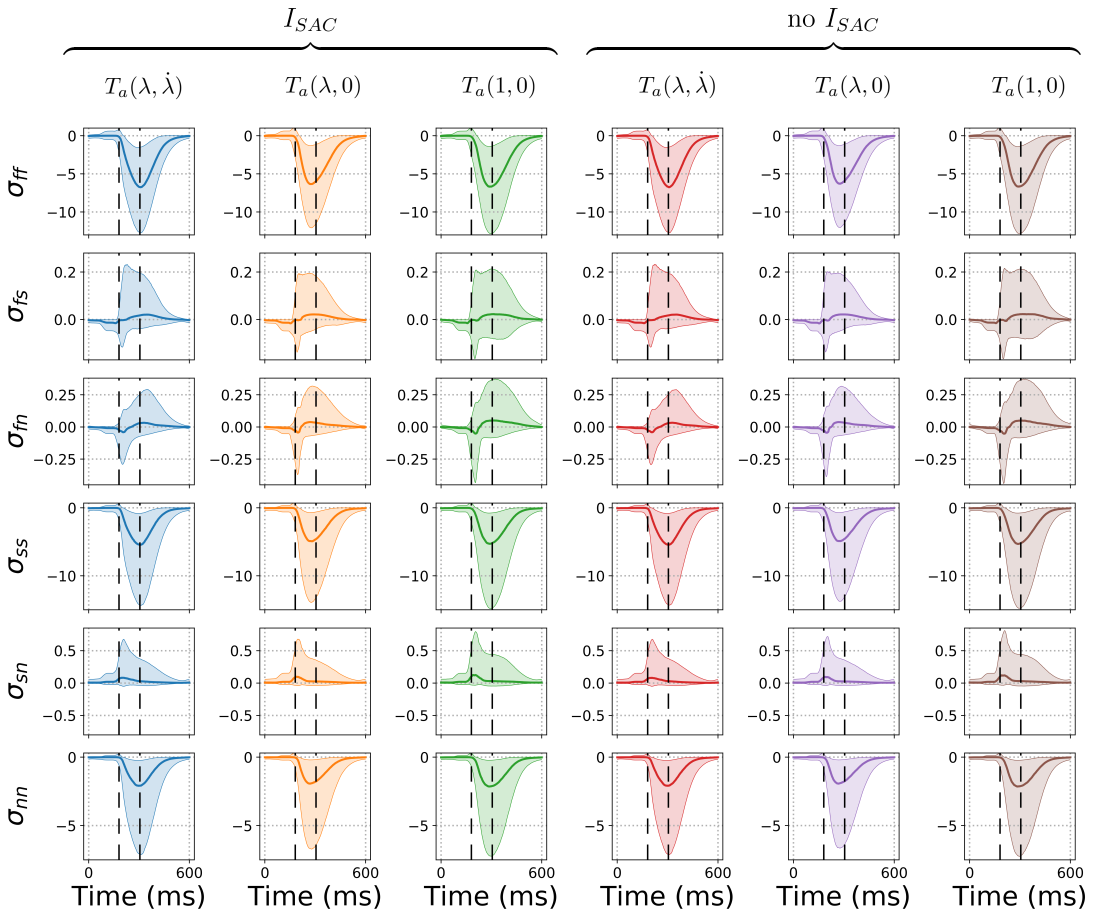

Figure 4: The stress distribution projected at the fiber, sheet and normal direction of the fibers of a slice at a cross section of the papillary muscle level of the LV over time. The ejection phase is indicated between the black dashed lines. The shaded area indicates the interquartile range while the solid line is the median.

different trigger levels of the SACs. At trigger levels of 8% and 7% the effect is visible on the depolarization times, and we get premature excitations at trigger levels of 6% and 5% which appear to balance the effect of the LBBB pathology.

This is also reflected in the stroke volume, which increases slowly for trigger levels up to 8% (0.2 mL changes). For higher sensitivity of the SACs, the stroke volume increases much faster until it reaches the healthy stroke volume at 5% trigger level. A rather interesting effect is the reduction of the maximal stretch ratio as the sensitivity of the SACs to stretch increases. In particular, the effect of the LBBB on the maximal stretch is not evident anymore for trigger levels of 6% and 5%.

6 Cardiac Resynchronization Therapy

Further simulations were performed to explore the effect of the MEF feedback on the CRT treatment for the LBBB pathology. The resulting PV-loops appear in figure 9. There is an improvement in the stroke volume in all cases when compared to the LBBB pathology. In particular, a larger difference in the stroke volume appears in the absence of the SACs, ranging from 0.9 mL to 1.8 mL.

Additionally, the depolarization times for all six models appear in figure 10. The changes in the depolarization times are minor in the absence of SACs. The SACs affect the depolarization times with apparent reduction in the activation times at the apex of the LV and near the base of the RV.

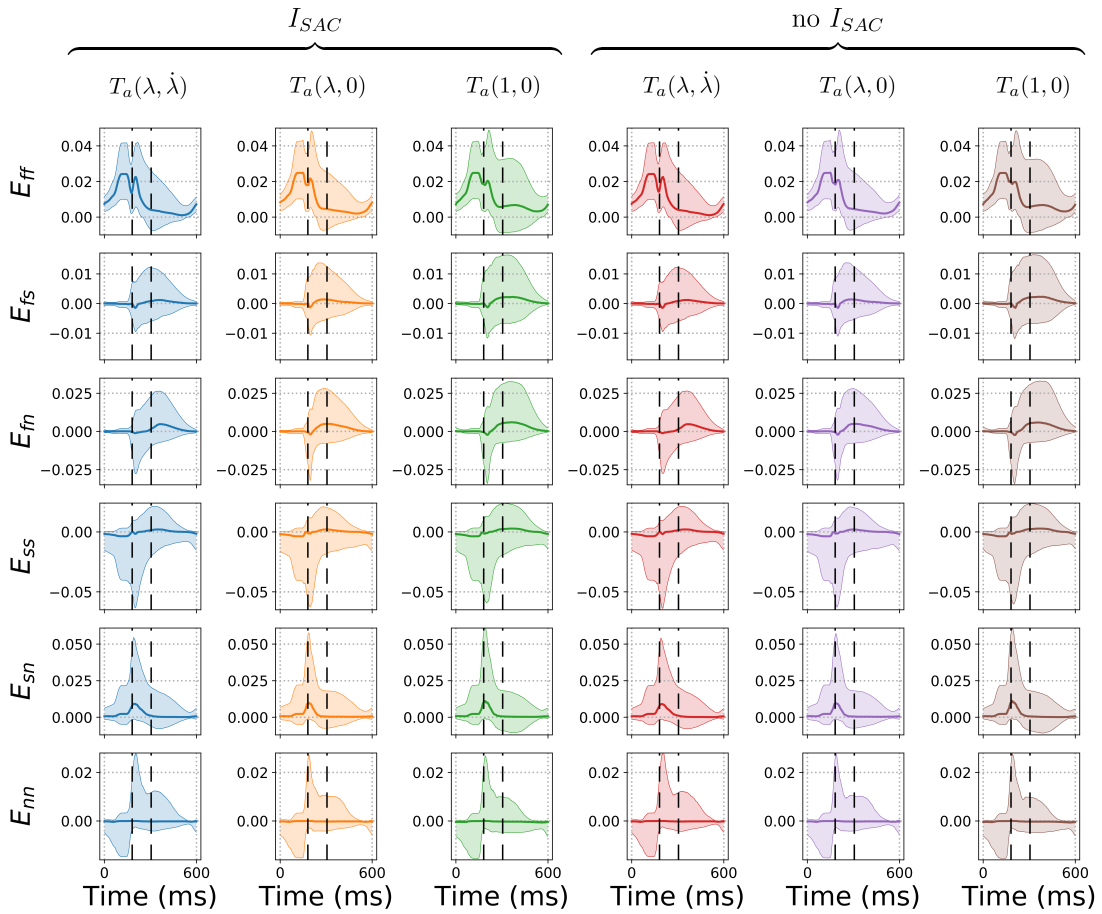

Figure 5: The strain distribution projected at the fiber, sheet and normal direction of the fibers of a slice at a cross section of the papillary muscle level of the LV over time. The ejection phase is indicated between the black dashed lines. The shaded area indicates the interquartile range while the solid line is the median.

7 Strong coupling of EP and active tension

We further explore the effect of the two-way coupling between the ionic and the active tension models at a cellular level. The first strongly coupled model that involved the Ten Tusscher model has been introduced by [2] and used the Ekatarinburg-Oxford active tension model [11]. In our approach, we introduce a strongly coupled Ten Tusscher [13] and the active tension model by Land et al. [5]. In particular, we modify the rate of change of the buffered calcium concentration of the Ten Tusscher model [13] to split the contributions of the troponin and calmodulin, similar to [6]. This results in the replacement of the equation

$$\begin{aligned} \frac{dCa_i}{dt} &= \beta_{Ca_i}\left(-\frac{I_{bCa} + I_{pCa} - 2I_{NaCa}}{2V_cF} + \frac{V_{sr}}{V_c}(I_{leak} - I_{up}) + I_{xfer}\right), \\ \beta_{Ca_i} &= \frac{1}{1 + \frac{Buf_c K_{bufc}}{(K_{bufc}+Ca_i)^2}}, \end{aligned}$$

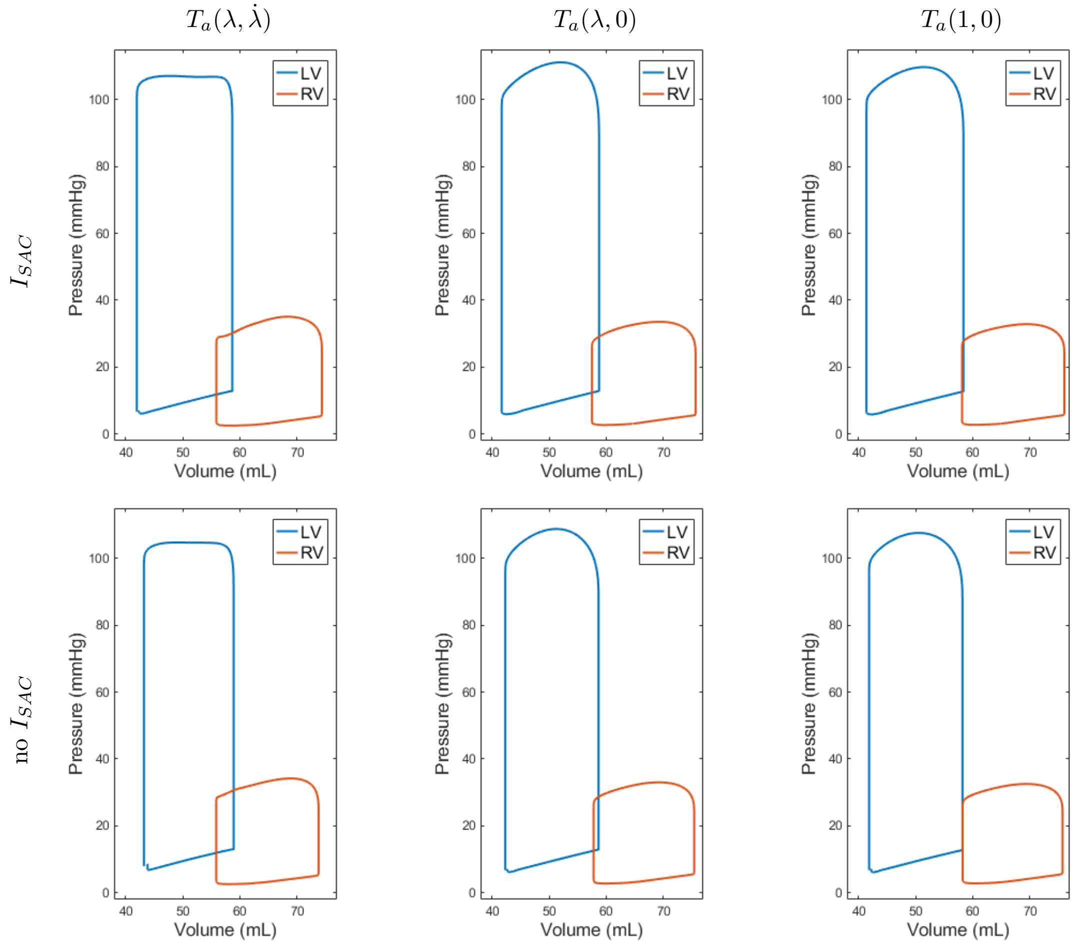

Figure 6: The PV-loops of the simulated heartbeats for different combinations of the MEF mechanisms for the LBBB pathology. The simulations without length dependence in the active tension model are re-tuned to the maximal tension $T_{ref} = 70$.

to the corresponding with the splitting of calmodulin and troponin in [9]

$$\begin{aligned}
\frac{dCa_i}{dt} &= \beta_{Ca_i}\left(-\frac{I_{bCa} + I_{pCa} - 2I_{NaCa}}{2V_cF} + \frac{V_{sr}}{V_c}(I_{leak} - I_{up}) + I_{xfer}\right), \\
\beta_{Ca_i} &= \frac{1}{1 + \frac{CMDN_{max}K_{m,CMDN}}{(K_{m,CMDN}+Ca_i)^2} + \frac{TRPN_{max}K_{m,TRPN}}{(K_{m,TRPN}+Ca_i)^2}}.
\end{aligned}$$

This modification is capable of accommodating a strong coupling, using the troponin equation from the active tension model [5]. In particular, a strong coupled model would take the form

$$\begin{aligned}
\frac{dCa_i}{dt} &= \beta_{Ca_i}\left(-\frac{I_{bCa} + I_{pCa} - 2I_{NaCa}}{2V_cF} + \frac{V_{sr}}{V_c}(I_{leak} - I_{up}) + I_{xfer} - TRPN_{max}\frac{dCaTRPN}{dt}\right), \\
\beta_{Ca_i} &= \frac{1}{1 + \frac{CMDN_{max}K_{m,CMDN}}{(K_{m,CMDN}+Ca_i)^2}}.
\end{aligned}$$

In the equations above, I_{bCa} is the background calcium current, I_{pCa} is the sarcolemmal calcium pump current, I_{NaCa} is the sodium potassium exchange current, V_c is the cytoplasmic volume, F the Faraday constant, V_{sr} the sarcoplasmic reticulum (SR) volume, I_{leak} the SR calcium leak current, I_{up} the SR calcium pump current, I_{xfer} the diffusive current between the diadic subspace

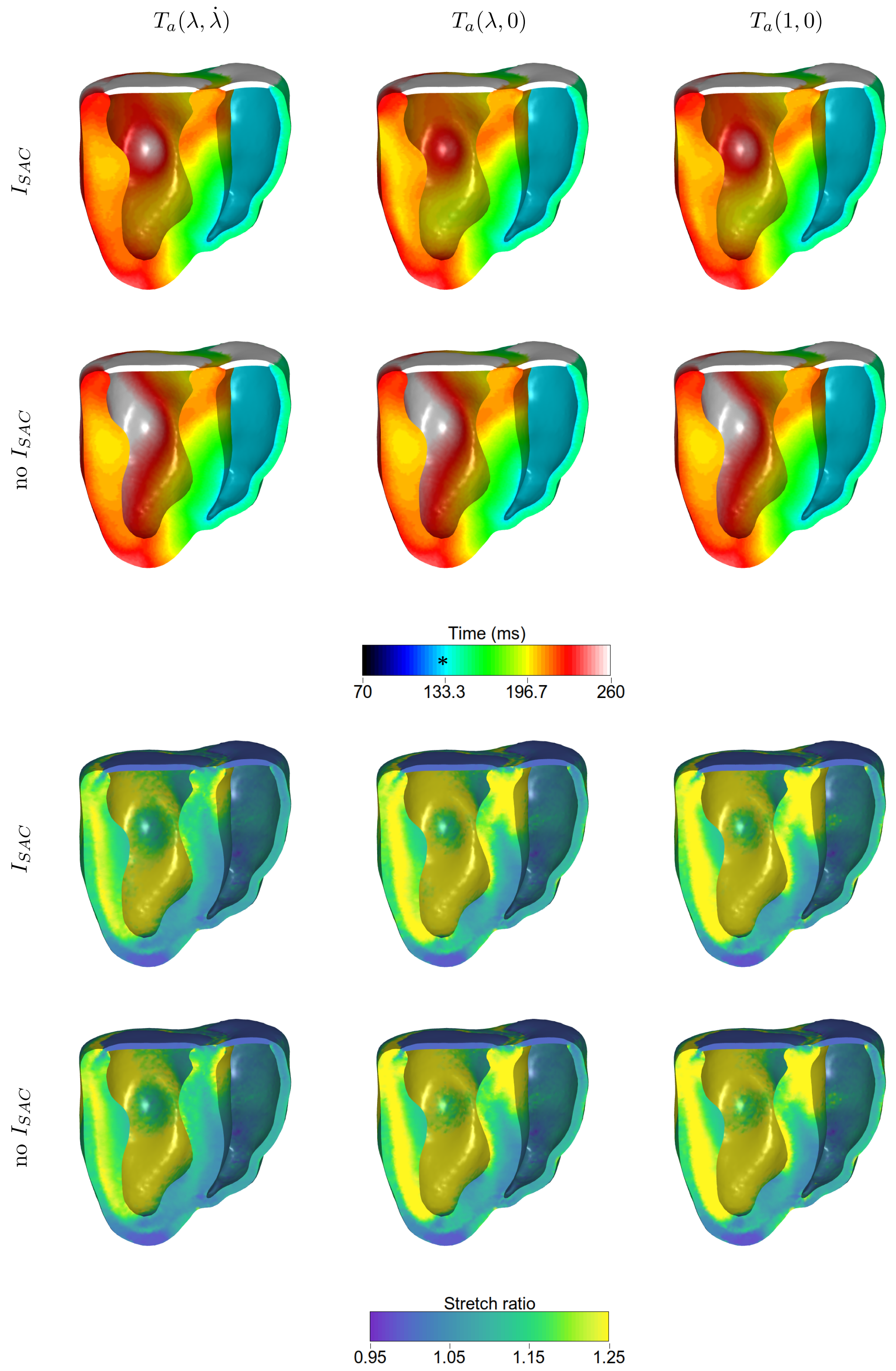

Figure 7: The depolarization times and the maximum stretch ratio of the simulated heartbeats for different combinations of the MEF mechanisms for the LBBB pathology. The $*$ indicates the onset of the ventricular depolarization at 130 ms.

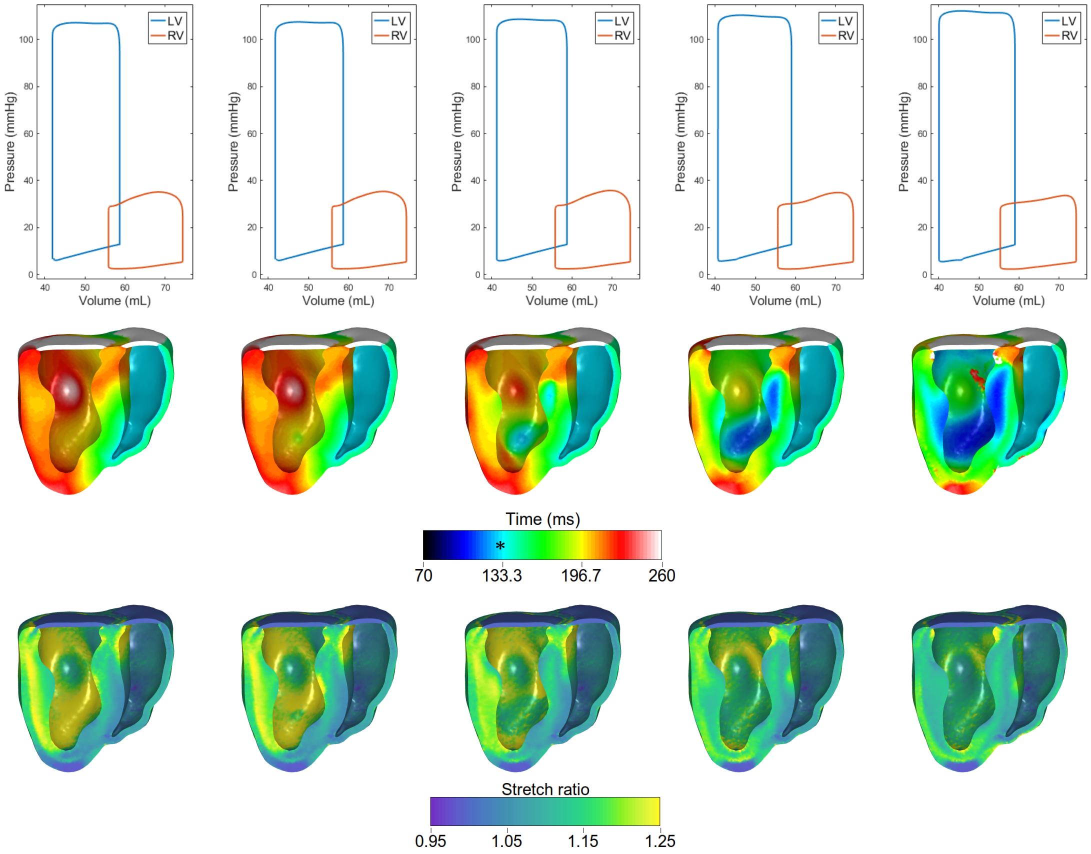

Figure 8: The effect of the SACs trigger level in the PV-loops, the depolarization times and the maximal stretch ratio in the presence of LBBB over a heartbeat. The $*$ indicates the onset of the ventricular depolarization at 130 ms.

and bulk cytoplasm, Buf_c the total cytoplasmic buffer concentration, K_{bufc} the calcium half-saturation constant for cytoplasmic buffer, $CMDN_{max}$ the maximum calmodulin, $K_{m,CMDN}$ the calmodulin half-saturation constant buffer, $TRPN_{max}$ the maximum troponin and $K_{m,TRPN}$ the troponin half-saturation constant buffer.

At first, we explore the impact of the modification on the coupling of the ionic and active tension models. Figure 11 shows the potential and the intracellular calcium using the original weak coupling, the modified weak coupling and the modified strong coupling. The parameters of the modified calcium buffer equation are taken from [9] and the maximal I_{CaL} conductance G_{CaL} is scaled by $2/3$ (see Table 2) to obtain comparable peak calcium as in the original weakly coupled model.

To assess the impact of the stretch in the strongly coupled ionic and active tension model, we explore the effect of a constant stretch ranging from $0.9-1.2$ on the intracellular calcium, troponin, tension and potential (see figure 12).

Finally, we consider the strongly coupled model and we include SACs to estimate the impact on the calcium, the potential, the troponin and the tension. We apply the stretch pattern observed at the endocardium, midwall and epicardium of the LV (figure 2 of the main manuscript) on the fully coupled model at steady state. To ensure the continuity of the stretch, we rescaled the stretch to the initial value of 1.

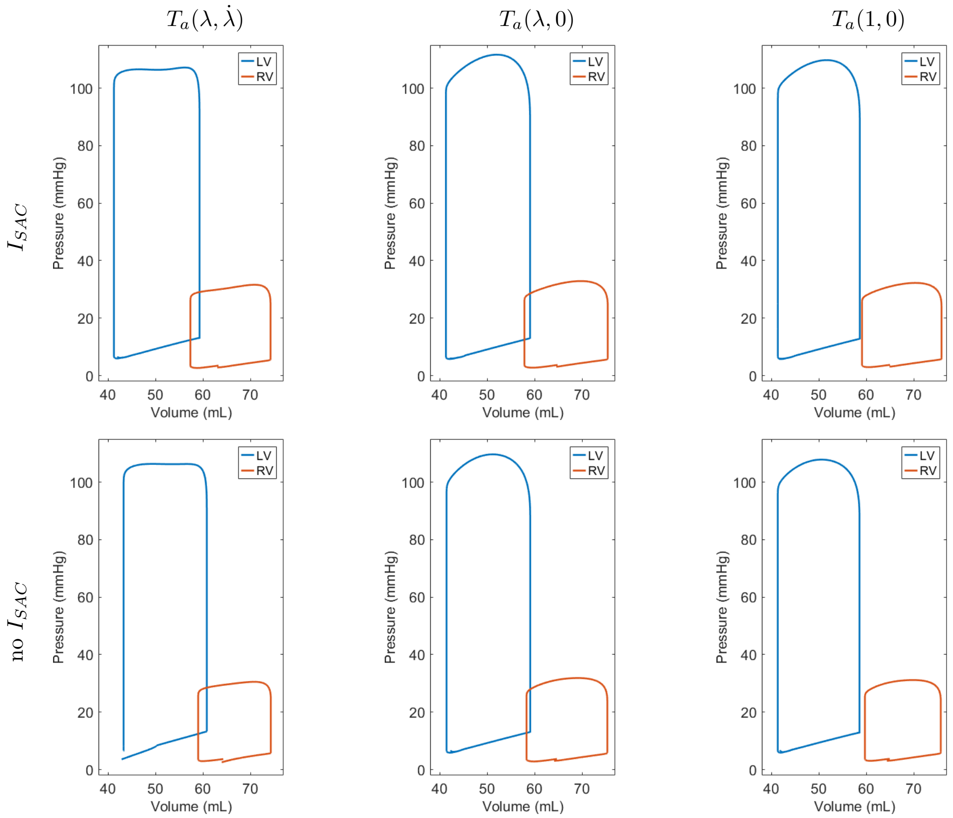

Figure 9: The PV-loops of the simulated heartbeats for different combinations of the MEF mechanisms for the CRT treatment. The simulations without length dependence in the active tension model are re-tuned to the maximal tension $T_{ref} = 70$.

References

[1] Christoph M Augustin, Matthias AF Gsell, Elias Karabelas, Erik Willemen, Frits W Prinzen, Joost Lumens, Edward J Vigmond, and Gernot Plank. A computationally efficient physiologically comprehensive 3d–0d closed-loop model of the heart and circulation. *Computer methods in applied mechanics and engineering*, 386:114092, 2021.

[2] Nathalie A Balakina-Vikulova, Alexander Panfilov, Olga Solovyova, and Leonid B Katsnelson. Mechano-calcium and mechano-electric feedbacks in the human cardiomyocyte analyzed in a mathematical model. *The Journal of Physiological Sciences*, 70(1):1–23, 2020.

[3] Chesapeake veterinary cardiology associates (cvca) manual of echocardiography. `https://www.cvcavets.com/documents/ManualofEchocardiography.pdf`. Accessed: 2022-04-07.

[4] Paul A. Iaizzo, editor. *Handbook of Cardiac Anatomy, Physiology, and Devices*. Springer International Publishing, 2015.

[5] Sander Land, So-Jin Park-Holohan, Nicolas P. Smith, Cristobal G. dos Remedios, Jonathan C. Kentish, and Steven A. Niederer. A model of cardiac contraction based on novel measurements of tension development in human cardiomyocytes. *Journal of Molecular and Cellular Cardiology*, 106:68–83, may 2017.

[6] F Levrero-Florencio, F Margara, E Zacur, A Bueno-Orovio, ZJ Wang, A Santiago, J Aguado-Sierra, G Houzeaux, V Grau, D Kay, et al. Sensitivity analysis of a strongly-coupled human-based electromechanical cardiac model: Effect of mechanical parameters on physiologically

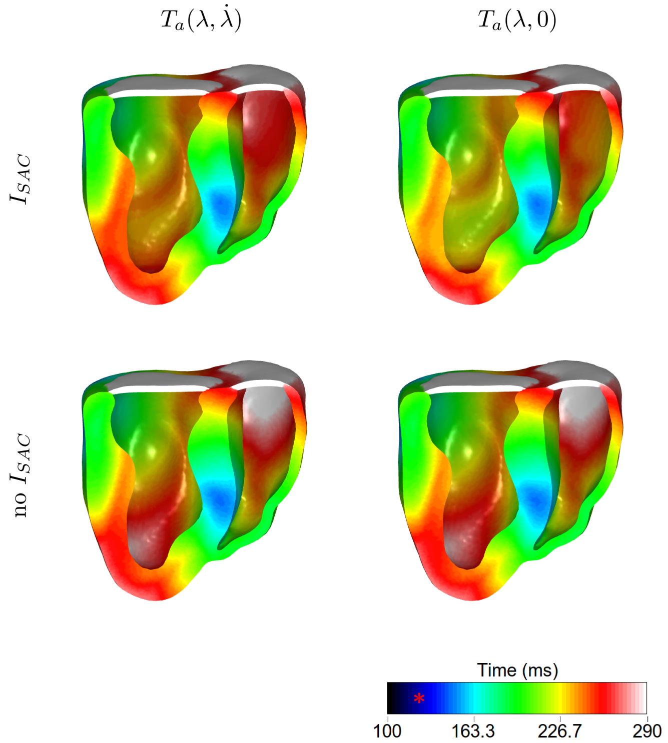

Figure 10: The depolarization times of the simulated heartbeats for different combinations of the MEF mechanisms for the CRT treatment of the LBBB pathology. The $*$ indicates the onset of the ventricular depolarization from the CRT leads at 120 ms.

relevant biomarkers. *Computer Methods in Applied Mechanics and Engineering*, 361:112762, 2020.

[7] Lili Liu, Bruce Tockman, Steven Girouard, Joseph Pastore, Greg Walcott, Bruce KenKnight, and Julio Spinelli. Left ventricular resynchronization therapy in a canine model of left bundle branch block. *American Journal of Physiology-Heart and Circulatory Physiology*, 282:H2238–H2244, 6 2002.

[8] Aurel Neic, Fernando O Campos, Anton J Prassl, Steven A Niederer, Martin J Bishop, Edward J Vigmond, and Gernot Plank. Efficient computation of electrograms and ecgs in human whole heart simulations using a reaction-eikonal model. *Journal of computational physics*, 346:191–211, 2017.

[9] Thomas O'Hara, László Virág, András Varró, and Yoram Rudy. Simulation of the undiseased human cardiac ventricular action potential: model formulation and experimental validation. *PLoS computational biology*, 7(5):e1002061, 2011.

[10] Frits W. Prinzen, William C. Hunter, Bradley T. Wyman, and Elliot R. McVeigh. Mapping of regional myocardial strain and work during ventricular pacing: Experimental study using magnetic resonance imaging tagging. *Journal of the American College of Cardiology*, 33(6):1735–1742, 1999.

[11] Tatiana Sulman, Leonid B Katsnelson, Olga Solovyova, and Vladimir S Markhasin. Mathematical modeling of mechanically modulated rhythm disturbances in homogeneous and heterogeneous myocardium with attenuated activity of na+–k+ pump. *Bulletin of mathematical biology*, 70(3):910–949, 2008.

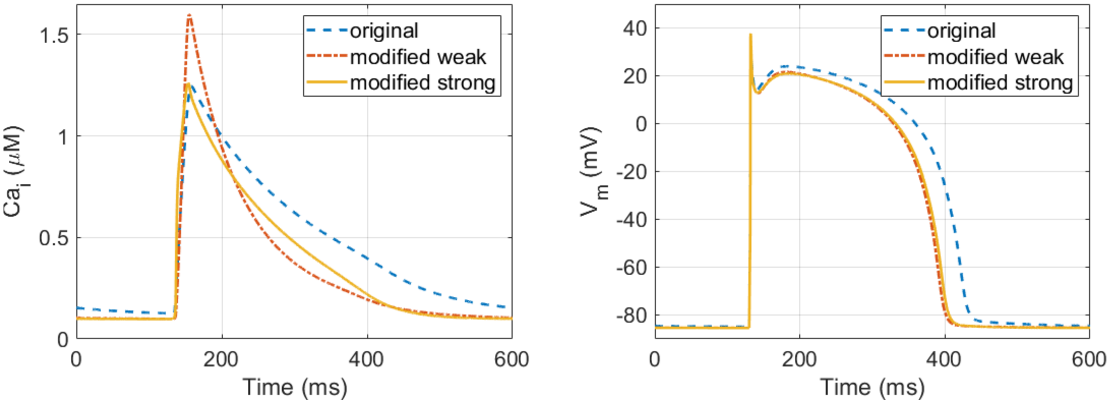

Figure 11: The effect on the intracellular calcium and potential of the proposed weak and strong coupling of the modified ionic and the active tension model against the original weak coupling used in the 3D simulations.

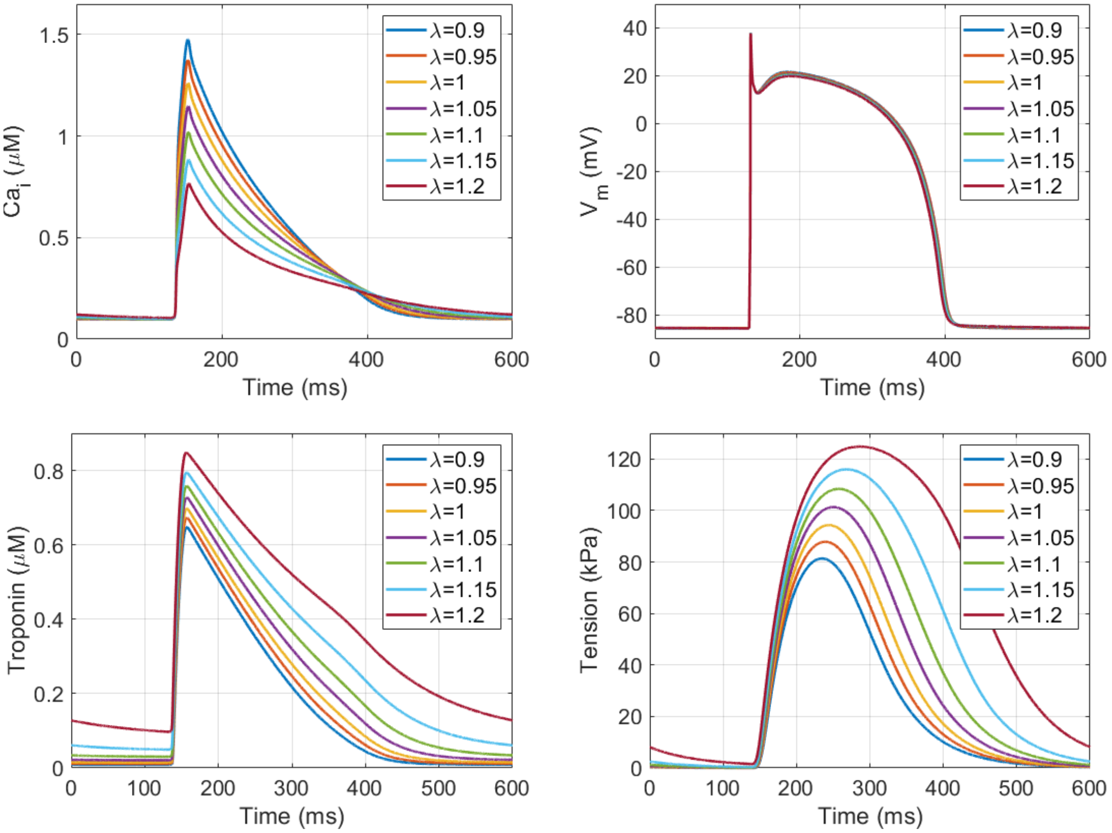

Figure 12: The intracellular calcium, potential, troponin and tension of the strongly coupled model for different applied constant stretch.

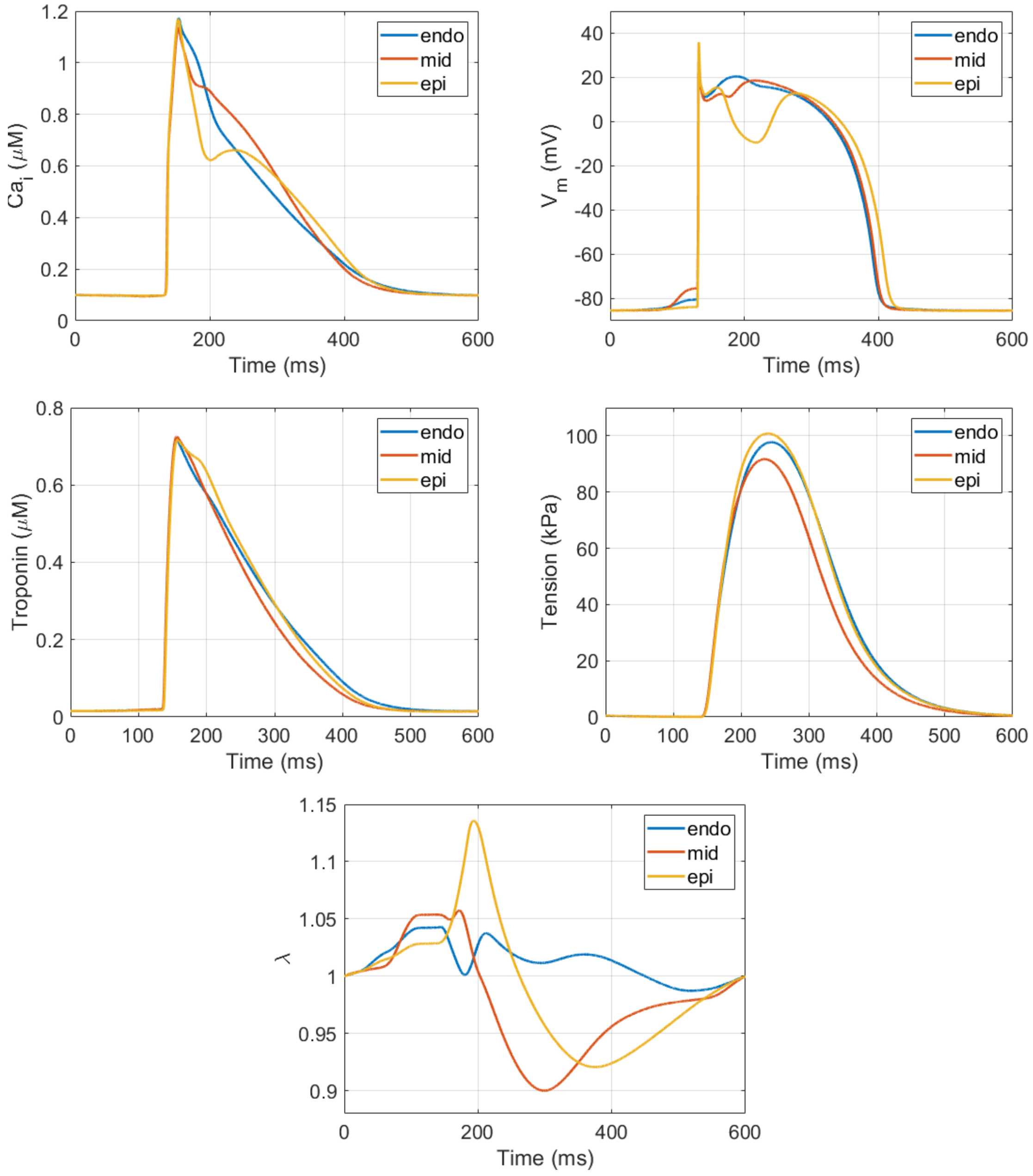

Figure 13: The intracellular calcium, potential, troponin, tension and stretch of the fully coupled model (strongly coupled model with SACs) for three different stretch patterns as observed at the endocardium (endo), midwall (mid) and epicardium (epi) of the LV in the 3D model, rescaled to ensure continuity of the stretch.

[12] Pasi Tavi, Chunlei Han, and Matti Weckström. Mechanisms of stretch-induced changes in [Ca2+] i in rat atrial myocytes: role of increased troponin C affinity and stretch-activated ion channels. *Circulation research*, 83(11):1165–1177, 1998.

[13] K. H. W. J. ten Tusscher and A. V. Panfilov. Alternans and spiral breakup in a human ventricular tissue model. *American Journal of Physiology-Heart and Circulatory Physiology*, 291(3):H1088–H1100, sep 2006.
